# Metabolites with anti-inflammatory activity from the mangrove endophytic fungus *Fusarium decemcellulare* DQ-28

**DOI:** 10.1039/d6ra05011e

**Published:** 2026-07-13

**Authors:** Ruxue Mu, Junhao Zhu, Zhongqian Xue, Yang Wang, Meng Zhang, Xinyao Chu, Jianning Dong, Ge Zou, Yan Chen

**Affiliations:** a School of Pharmacy, Anhui Medical University Hefei China cychemistry@ahmu.edu.cn; b School of Pharmacy, Xianning Medical College, Hubei University of Science and Technology Xianning China zoug5@hbust.edu.cn; c The First Affiliated Hospital of Anhui Medical University Hefei China

## Abstract

The mangrove-derived fungus *Fusarium decemcellulare* DQ-28 isolated from *Kandelia obovata* Sheue & *et.al*. and cultured on a solid rice medium yielded five new secondary metabolites, including one diphenolic derivative decemcellulin A (1), one butenolide derivative decemcellulin B (2), two furo-α-pyrone derivatives (±)- decemcellulin C (3), and one sesquiterpene derivative decemcellulene A (4). Their structures and absolute configurations were established by NMR and HRESIMS spectra, electronic circular dichroism (ECD) calculations and single-crystal X-ray diffraction experiments. Compound 1 exhibited potent anti-inflammatory activity with an IC_50_ value of 19.61 ± 0.14 µM. Western blot analysis revealed that 1 effectively suppressed the protein level of inducible nitric oxide synthase (iNOS) induced by LPS in RAW264.7 cells.

## Abstract 2

Five new secondary metabolites were isolated from *Fusarium decemcellulare* DQ-28. Among them, diphenolic derivative (1) exhibits potent anti-inflammatory activity by suppressing LPS-induced iNOS in RAW264.7 cells.

## Graphical abstract

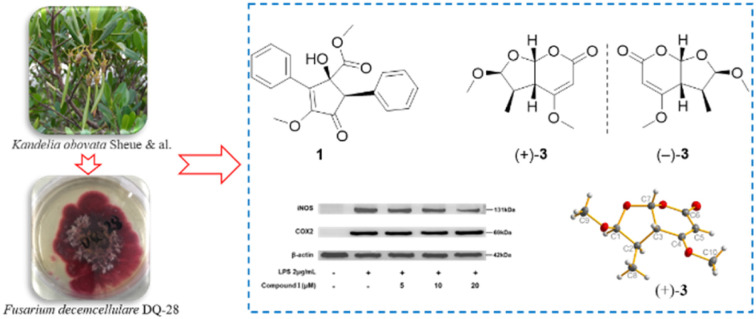

## Introduction

The genus *Fusarium* is a highly diverse fungal group, comprising over one thousand species.^1^ While most are important plant pathogens, some function as endophytes in symbiosis with host plants. Representative members of this genus include *Fusarium decemcellulare*, *Fusarium oxysporum*, *Fusarium graminearum*, *Fusarium solani*, *Fusarium verticillioides*, and *Fusarium incarnatum*, among others.^2,3^ Plant-derived endophytic *Fusarium* strains produce a rich array of secondary metabolites, including polyketides, alkaloids, and terpenoids,^4^ many of which exhibit cytotoxic,^5^ anti-inflammatory,^6^ antiviral,^7^ anti-atherosclerosis,^8^ antibacterial,^9,10^ antiproliferative^11^ and antioxidant^12^ activities. Mangrove-derived endophytic fungi exhibit rich species diversity, and their secondary metabolites are characterized by novel structures and remarkable biological activities.^13–18^

**Fig. 1:**
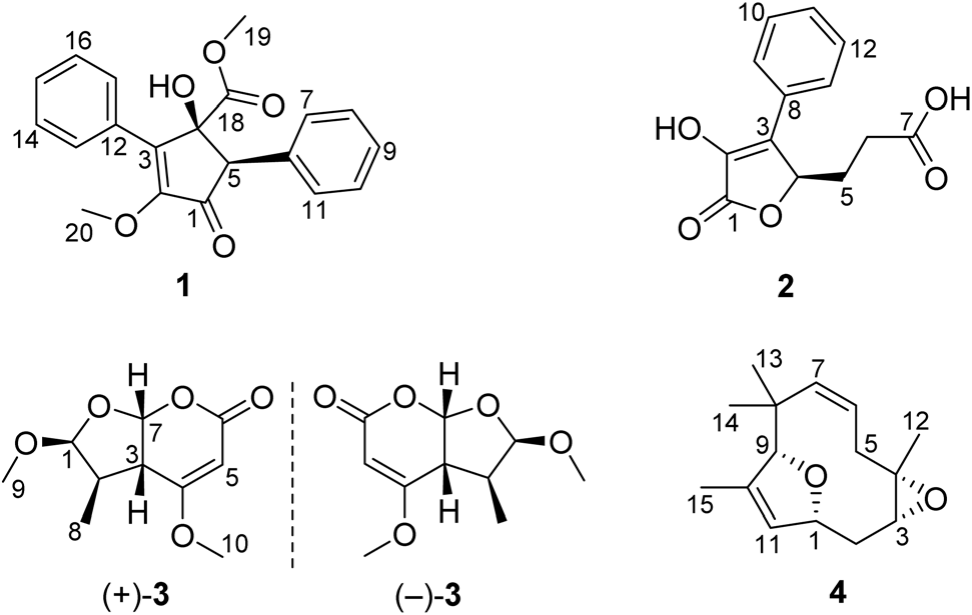
The structures of 1–4.

In our ongoing search for bioactive natural products from mangrove-derived fungi, the fungus *Fusarium decemcellulare* DQ-28 was found to form distinctive red colonies on potato dextrose agar (PDA) plates, and abundant secondary metabolites were produced. Promising anti-inflammatory activity was observed in the crude extract obtained from preliminary fermentation. Following scale-up fermentation in rice medium, five new secondary metabolites, including one diphenyl derivative decemcellulin A (1), one butenolide derivative decemcellulin B (2), two furo-α-pyrone derivatives (±)- decemcellulin C (3), one sesquiterpene derivative decemcellulene A (4) were isolated from the mangrove endophytic fungus *Fusarium decemcellulare* DQ-28. The anti-inflammatory effects of all compounds were evaluated against lipopolysaccharide (LPS)- activated RAW264.7 macrophages. Compound 1 exhibited potent anti-inflammatory activity with IC_50_ value of 19.61 ± 0.14 µM. Western blot analysis revealed that compound 1 exerted an anti-inflammatory effect by effectively inhibiting the protein expression level of inducible nitric oxide synthase (iNOS) in lipopolysaccharide-induced mouse mononuclear macrophages RAW264.7. Herein, we describe the isolation, structural elucidation, and biological evaluation of the obtained compounds (Fig. 1).

## Results and discussion

### Structure elucidation

**Fig. 2:**
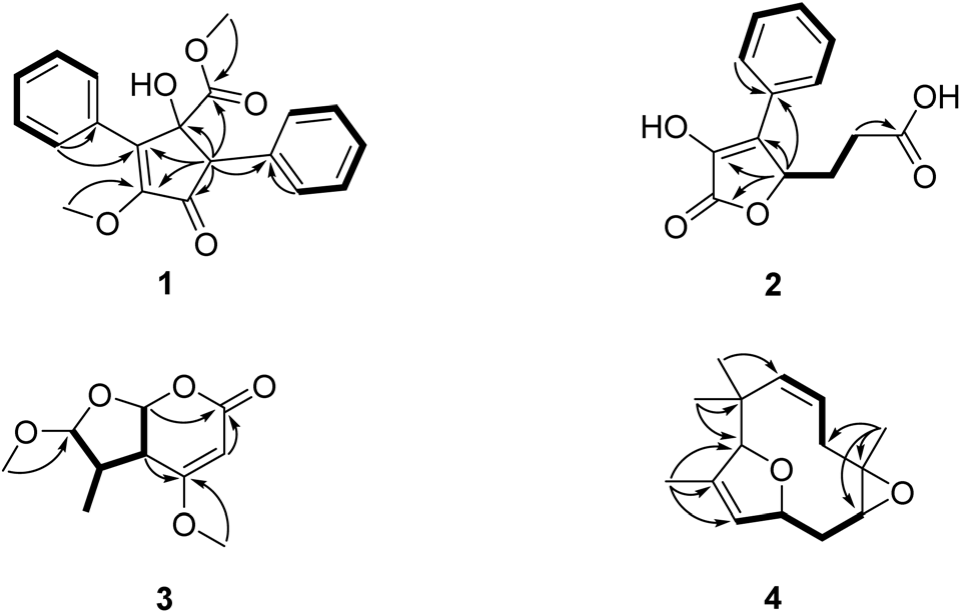
The key ^1^H–^1^H COSY and HMBC correlations for 1–4.

**Fig. 3:**
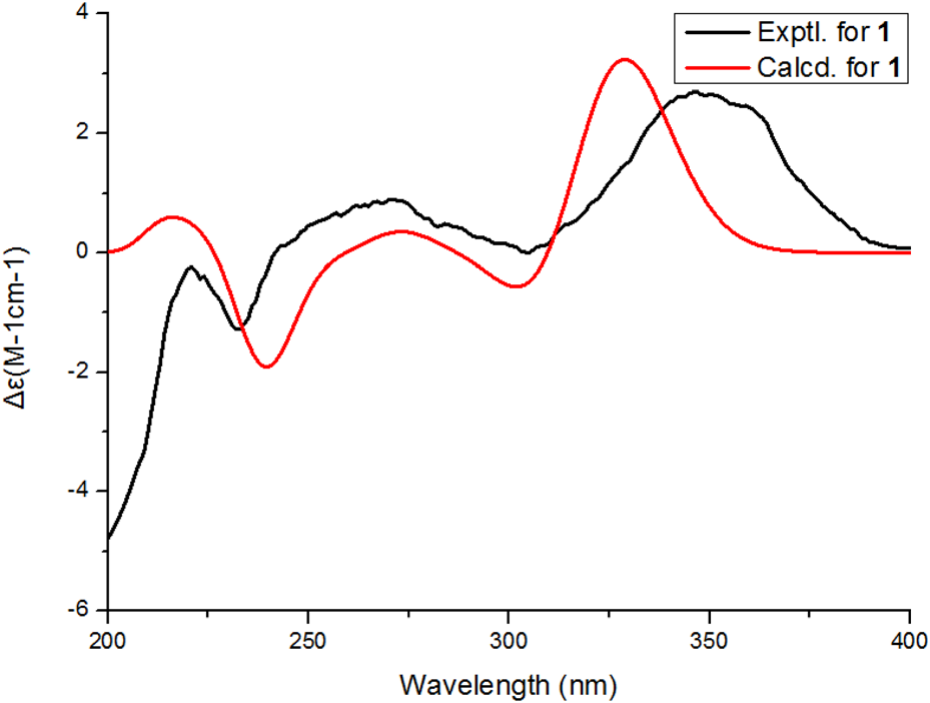
Experimental and calculated ECD spectra of 1 in MeOH.

Compound 1 isolated as a white solid, had a chemical formula of C_20_H_18_O_5_ and twelve degrees of unsaturation by analysis of HRESIMS spectrum (Fig. S6). The ^1^H-NMR spectrum (Table 1) revealed the presence of two methoxy groups at *δ*_H_ 3.77 (s, 3H) and 4.15 (s, 3H), ten aromatic proton signals at *δ*_H_ 7.15 (m, 2H), 7.40 (m, 6H), and 7.82 (m, 2H), indicating that compound 1 contain two monosubstituted benzene ring segments. The ^13^C NMR and HSQC spectral displayed 20 carbon signals, including two methoxy groups, eleven methines (ten sp^2^ carbons), and seven non hydrogenated carbons (four sp^2^ carbons and two carbonyls). The HMBC correlations (Fig. 2) from H-13 to C-12 and C-3, from H-5 to C-1, C-2, C-3, C-4, C-6, and C-18, and H-11 to C-6, revealed that the two monosubstituted benzene rings were replaced at C-3 and C-5 of the five-membered ring nucleus. The HMBC cross peaks from H_3_-19 to C-18 and from H_3_-20 to C-2 supported that the two methoxy groups were assigned to C-2 and C-18. Thus, the planar structure of compound 1 was determined as shown in Fig. 1. While, no correlation was observed in the NOESY spectra (Fig. S7). The relative configuration at C-4 and C-5 were determined by the ^13^C NMR calculation. Two possible relative configurations (4S*5R*)-1a and (4S*5R*)-1b were performed using the GIAO method at the mPW1PW91/SCRF/6-311+G(d,p)/PCM chloroform) level (Table S1). Finally, DP4+ probability 100% (all data) analysis confirmed that (4S*5R*)-1a configuration was the most probable structure, with a correlation coefficient *R*^2^ = 0.9987 (Fig. S27). Furthermore, the absolute configuration of compound 1 was confirmed as 4*S*,5*R* by the electronic circular dichroism (ECD) calculation (Fig. 3).

**Table 1:** ^1^H (500 MHz) and ^13^C (125 MHz) NMR data of 1 in CDCl_3_

1			1		
No	*δ*_C_, type	*δ*_H_ (*J* in Hz)	No	*δ*_C_, type	*δ*_H_ (*J* in Hz)
1	199.3, C		9	128.2, CH	7.40, m
2	153.4, C		12	131.8, C	
3	142.7, C		13, 17	128.6, CH	7.82, m
4	78.7, C		14, 16	128.8, CH	7.40, m
5	60.7, CH	4.01, s	15	129.6, CH	7.40, m
6	132.9, C		18	174.6, C	
7, 11	130.6, CH	7.15, d (6.4)	19	53.6, CH_3_	3.77, s
8, 10	128.7, CH	7.40, m	20	58.8, CH_3_	4.15, s

Compound 2 was isolated as a yellow solid. The HRESIMS data revealed its molecular formula as C_13_H_12_O_5_ with eight degrees of unsaturation. The ^1^H NMR spectrum (Table S2) of 1 showed the presence of five aromatic proton signals at *δ*_H_ 7.75 (2H, d, *J* = 7.5 Hz), 7.44 (2H, d, *J* = 7.5 Hz), and 7.35 (1H, d, *J* = 7.5 Hz). The ^13^C NMR and HSQC spectra revealed the coexistence of 13 carbon signals, including two methylenes, six methines (five sp^2^ carbons), and five non hydrogenated carbons (three sp^2^ carbons and two carbonyls). Analysis of the ^1^H–^1^H COSY correlations revealed two spin coupling systems (Fig. 2). The planar structure of 2 was further confirmed by the HMBC cross-peaks from H-4 to C-1, C-2, C-3 and C-8, from H-6 to C-7, and from H-11 to C-8. Comparison of the ^13^C NMR spectra revealed similarities between 2 and WF-3681, which was identified as racemate from *Chaetomella raphigera*.^19^ In this research, 2 was isolated as optically pure monomeric compound. The configuration of 2 was confirmed as 4*R* by ECD calculation (Fig. S28), with a specific rotation value of [*α*] +25.6 (*c* = 0.23, MeOH).

**Fig. 4:**
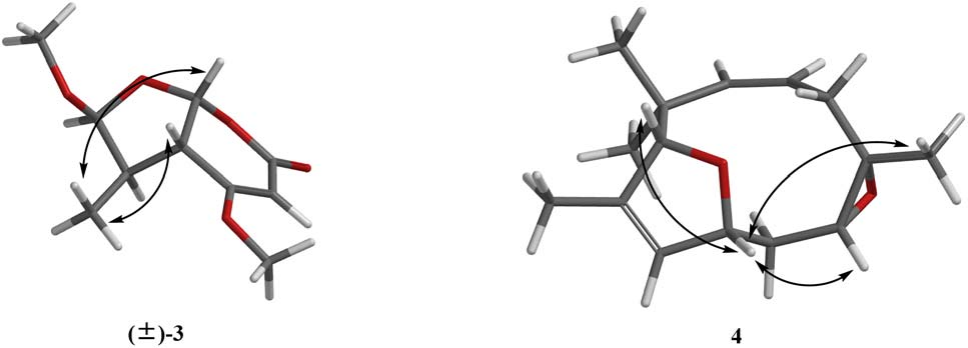
NOESY correlations of 3–4.

**Fig. 5:**
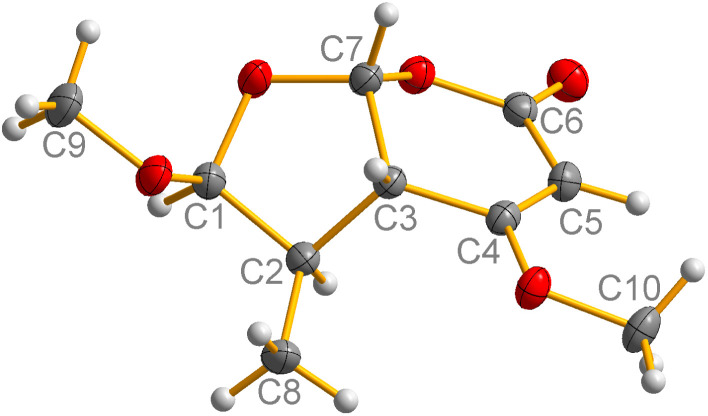
X-ray crystallographic analysis of (+)-3.

Compound (±) -3 was isolated as yellow crystals. It has achemical formula of C_10_H_14_O_5_ and four degrees of unsaturation derived from the HRESIMS data. The ^1^H and ^13^C NMR data (Table S2) of (±)-3 showed three methyls (two oxygenated), five methines (one olefinic and two oxygenated), and two non-hydrogenated carbons (one carbonyl). The planar structure of (±) -3 was supported by the ^1^H–^1^H COSY correlation (Fig. 2) of H-1/H-2(/H_3_-8)/H-3/H-7 along with HMBC cross-peaks (Fig. 2) from H-9 to C-1, from H-3 and H_3_-10 to C-4, and from H-7 and H-5 to C-6. Notably, both the optical rotation and CD maximum of (±)-3 were close to zero suggesting that (±)-3 was a racemic. Subsequently, it was separated by a chiral column (*S*-Chiral A, 5 µm) to obtain compounds (+)-3 and (−)-3 (Fig. S29). The relative configuration was determined by NOESY correlations (Fig. 4). Subsequently, the crystal structure of (+)-3 was successfully obtained in methanol solvent (Fig. 5), which confirmed the structure of (+)-3 and showed that its absolute configuration was 1*S*, 2*R*, 3*S*, 7*R*. Therefore, the configuration of (−)-3 was determined as 1*R*, 2*S*, 3*R*, 7*S* (Fig. S28).

Compound 4 was isolated as a yellow oil. The molecular formula was determined to be C_15_H_22_O_2_ based on HRESIMS data. The ^1^H NMR spectrum (Table S3) included four methyl signals at *δ*_H_ 1.31 (s), 1.16 (s), 1.07 (s), and 1.75 (s), three olefin proton signals at *δ*_H_ 5.38 (ddd, *J* = 15.7, 10.9, 4.8 Hz, H-6), 5.27, d (10.2), and 5.05 (d, *J* = 15.65 Hz, H-7). The ^13^C NMR data (Table S3) indicated 15 carbons including four methyls, two methylenes, six methines (three olefinic and three oxygenated), and three non-hydrogenated carbons (one olefinic). The ^1^H–^1^H COSY correlations (Fig. 2) of H-5/H-6/H-7, and H-11/H^−1^-1/H_2_-2/H_2_-3 together with HMBC cross-peaks from H_3_-12 to C-3, C-4, and C-5, from H_3_-13 to C-7, H_3_-14 to C-8 and C-9, and from H_3_-15 to C-9, C-10, and C-11, established the planar structure of 4. Thereafter, the relative configuration of 4 was determined by the NOESY correlations (Fig. 4). The absolute configuration was confirmed as 1*R*, 3*R*, 4*S*, 9*R* by the experimental and calculated ECD spectra (Fig. S28).

### NO inhibitory activity against RAW264.7 cells

**Fig. 6:**
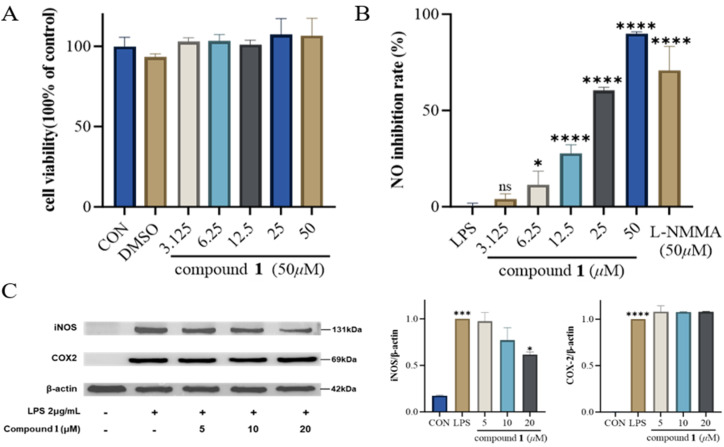
(A) Cell viabilities of compound 1. (B) Effects of compound 1 and L-NMMA on NO production in LPS-induced RAW264.7 cells. (C) Protein levels of COX-2, iNOS, and β-actin by western blot. All presented data are expressed as the mean ± SD, with *n* = 3. Ns: no significant, (*) *p* < 0.05, (***) *p* < 0.001, and (****) *p* < 0.0001 *versus* the LPS group.

The inhibitory effects of all compounds on nitric oxide (NO) production were evaluated in lipopolysaccharide (LPS)-induced RAW 264.7 murine macrophages. As shown in Fig. 6A, compound 1 exhibited no cytotoxicity against RAW264.7 macrophages at a concentration of 50 µM by CCK-8 assay. Subsequent NO assay results (Fig. 6B) revealed that compound 1 significantly suppressed NO expression in a dose-dependent manner, with IC_50_ value of 19.61 ± 0.14 µM; a positive control, *N*^ω^-methyl-l-arginine (L-NMMA), exhibiting IC_50_ value of 17.75 ± 0.55 µM. While, compounds 2–4 did not exhibit significant NO inhibitory activity at the concentration of 50 µM. Western blot analysis revealed that compound 1 selectively downregulated iNOS protein expression in a dose dependent manner. Whereas it exerted no significant effect on the protein expression of COX 2 (Fig. 6C). Marine-derived fungi are a promising source of structurally diverse and novel secondary metabolites with potent anti-inflammatory activity.^20^ For example, talaromeroterpenoid B from *Talaromyces* sp. JNQQJ-4 exhibits anti-inflammatory activity by inhibiting the NF-κB signaling pathway;^15^ epimicrosphazaphilone A from *Microsphaeropsis arundinis* P1B exerts anti-inflammatory effects *via* suppressing the ERK1/2 MAPK signaling pathway;^21^*cis*-resorcylide from *Penicillium* sp. HN20 produces anti-inflammatory activity by blocking the MAPK/ERK pathway;^22^ and asperthrin A from *Aspergillus* sp. FAZW0001 exerts anti-inflammatory effects through MAPK/NF-κB-mediated inhibition of the NLRP3 inflammasome.^23^

## Experimental

### General experimental procedures

Melting points were recorded on a SGW X-4 melting-point apparatus. The optical rotations were measured at 25 °C in MeOH using an MCP 300 polarimeter (Anton Paar). The UV spectra were recorded using a Shimadzu UV-1700 spectrometer (Shimadzu, Tokyo, Japan). IR spectra were obtained using KBr discs on a Nicolet Nexus 670 spectrophotometer. The CD spectra were obtained from a model 420SF CD spectrometer (Aviv Biomedical Inc). IR spectra were measured in KBr discs using a Bruker Avance 500 spectrometer. All NMR tests were performed using a Bruker Avance 500 spectrometer at room temperature. HRESIMS data were performed using a Thermo Scientific Q Exactive Orbitrap MS system. Column chromatography was performed using silica gel (200–300 mesh, Qingdao Marine Chemical) and Sephadex LH-20 (Pharmacia Sweden). Thin-layer chromatography (TLC) was performed on silica gel plates (HSGF254) from Qingdao Hai Yang Silica Company. Semi-preparative HPLC was performed on an Agilent 1260 Infinity II with Chiral chromatography column (OD-H, 5 µm, 4.6 × 250 mm; Daicel).

### Fungal material

The fungus was obtained from *Kandelia obovata Sheue* & al. Fresh branch from the Dongzhaigang Mangrove National Nature Reserve located in Hainan province of China in July 2022. The fungi were identified as *Fusarium decemcellulare* by amplifying DNA and analyzing ITS sequences.^24^ The sequence data of this strain was 100% homologous to that of *Fusarium decemcellulare* (KX788159.1), and the GenBank accession number was PZ448479. The fungus strain has been maintained at Anhui Medical University.

### Fermentation, extraction, and isolation

The fungal culture was inoculated onto solid rice medium. A total of 100 × 1L Erlenmeyer flasks were employed, each containing 100 g of unpolished rice and 90 mL of 0.3% seawater, followed by static incubation at 28 °C for 30 days. The whole fermentation culture was then extracted three times with methanol, affording 120.6 g of crude extract. The extract was then subjected to a silica gel (200–300 mesh) column with a stepwise gradient elution of petroleum ether (PE) and EtOAc (v/v, 100 : 0, 90 : 10, 80 : 20, 70 : 30, 60 : 40, 50 : 50, 40 : 60, 30 : 70, and 0 : 100), yielding ten fractions (Fr.1–Fr.10). Fr.4 was purified by silica gel CC (CH_2_Cl_2_/MeOH v/v, 92 : 8) to obtain compound 1 (5.2 mg) and subfractions (Fr.4.1–4.2). Fr.4.2 was purified using semipreparative HPLC (INA column, Hex/iPr v/v = 80 : 20, 1.5 mL min^−1^) to obtain compound 4 (3.5 mg, *t*_R_ = 8.6 min). Compound 2 (6.0 mg) was isolated by silica gel CC eluting with CH_2_Cl_2_/MeOH (95 : 5, v/v) from Fr.5. Fr.6 was subjected to Sephadex LH-20 eluting with CH_2_Cl_2_/MeOH (1 : 1, v/v) to afford three subfractions (Fr.6.1–6.3). Compounds (+)-3 (3.3 mg, *t*_R_ = 13.5 min) and (−)-3 (3.0 mg, *t*_R_ = 14.2 min) were separated using semipreparative HPLC (INA column, Hex/iPr v/v = 75 : 25) from Fr.6.2.

Decemcellulin A (1): white solid; [*α*] = 56.6 (*c* 0.12, MeOH); UV (MeOH) *λ*_max_ (log *ε*): 338(3.62), 276(3.35), 228(3.86) nm; ^1^H (500 MHz, CDCl_3_) and ^13^C NMR data (125 MHz, CDCl_3_), Table 1; HRESIMS *m*/*z* 337.10718 [M − H]^−^ (calcd for C_20_H_18_O_5_, 337.10720).

Decemcellulin B (2): yellow solid; [*α*] = 25.6 (*c* = 0.23, MeOH); UV (MeOH) *λ*_max_ (log *ε*): 218(3.23), 290(3.15) nm; ^1^H (500 MHz, CDCl_3_) and ^13^C NMR data (125 MHz, CDCl_3_), Table S2; HRESIMS *m*/*z* 271.05808 [M + Na]^+^ (calcd for C_13_H_12_O_5_Na, 271.05810).

Decemcellulin C (+)-3: mp 215–217 °C, recrystallized from methanol; colorless crystals; [*α*] +32.4 (*c* 0.12, MeOH); UV (MeOH) *λ*_max_ (log *ε*): 225(3.10), 270(2.65), 310(1.20) nm; ^1^H and ^13^C NMR (500 MHz, CDCl_3_) data, see Table S2; HRESIMS *m*/*z* 237.0734 [M + Na]^+^ (calcd for 237.0736, C_10_H_14_O_5_Na); compound (–)-3: colorless oil; [*α*] −40.2 (*c* 0.20, MeOH); demcellulene A (4): yellow oil; [*α*] = − 23 (*c* 0.25, MeOH); ^1^H (500 MHz, CDCl_3_) and ^13^C NMR data (125 MHz, CDCl_3_), Table S3; HRESIMS *m*/*z* 235.1691 [M + H]^+^ (calcd for C_15_H_22_O_2_, 235.1690).

### ECD calculation methods

The ECD calculation was carried out by the method outlined previously.^25,26^ Compound conformations were modified using the density functional technique in Gauss 09 at the B3LYP/6-31g (d) level. Then, theoretical calculations were performed at B3LYP/6-311g levels using TD-DFT.

## X-ray crystallographic analysis of (+)-3

Crystal data were collected from a colorless prism (0.3 × 0.2 × 0.1 mm^3^) at 150.0 K: C_10_H_14_NO_5_, *M*_W_ = 856.84, triclinic, space group P1, unit cell dimensions *a* = 4.7196(2) Å, *b* = 7.9364(4) Å, *c* = 27.8618(13) Å, *V* = 1043.61(8) Å3; *α* = 90.00°, *β* = 90.00°, *γ* = 90.00°, *Z* = 1, *ρ*_calc_ = 1.363 g cm^−3^, *F*(000) = 456.0. Data collection was performed on an Xcalibur Eos diffractometer with graphite monochromator, Ga Kα radiation. A total of 33 010 reflections measured, 10 383 unique (*R*_int_ = 0.0232) which were used in all calculations. The structure was solved with the SHELXS (G.M. Sheldrick, Acta Cryst, 2008), and refined by a full-matrix least-squares method on *F*^2^ by means of SHELXL (G.M. Sheldrick, Acta Cryst, 2008). The final *R* indices [*I* > 2*σ*(*I*)], *R*_1_ = 0.0267, *wR*_2_ = 0.0738; *R* indices [all data], *R*_1_ = 0.0273, *wR*_2_ = 0.0741. The Flack parameter value was −0.01(3). Crystallographic data for the structure of (+)-3 has been deposited with the Cambridge Crystallographic Data Centre and allocated the deposition number CCDC 2556838.

### Cell culture and treatment

Mouse macrophages RAW 264.7 were cultured in high glucose DMEM supplemented with 10% FBS and 1% penicillin streptomycin, and maintained at 37 °C under a humidified atmosphere of 5% CO_2_ and 95% humidity. Subsequently, cells were passaged 1 : 3 or 1 : 4, when confluence reached 80%.

### Cell viability assay

When cells reached the logarithmic growth phase, they were seeded into 96 well plates at a density of 1 × 10^4^ cells per well. After 24 h of incubation, the culture medium was removed and replaced with either drug containing or control basal medium. Five drug concentrations were applied: 50, 25, 12.5, 6.25, and 3.125 µM. Following another 16 h incubation, cell viability was evaluated using the CCK 8 assay. A mixture of basal DMEM and CCK 8 solution (10 : 1, v/v) was added to each well (110 µL per well), and the plates were incubated at 37 °C for 1–4 h. Absorbance was then measured at 450 nm using a Thermo Fisher microplate reader. The formula (cell viability = (OD_compound_ − OD_blank_)/(OD_Con_ − OD_blank_) × 100%) has been used to calculate the cell viability.^27^

### Anti-inflammatory assay

Following the same seeding procedure, the culture medium was removed and replaced with basal medium containing test compounds and LPS, or without them, for intervention. Experimental groups were set as follows: a normal control group, an LPS model group, and drug treatment groups at concentrations of 50, 25, 12.5, 6.25, and 3.125 µM. After 24 h of incubation, cell supernatants were collected. NO levels were determined using an NO detection kit. For each well, 50 µL of supernatant was mixed with 50 µL of Reagent I (sulfanilamide) and 50 µL of Reagent II (*N*(1 naphthyl) ethylenediamine dihydrochloride) successively. After full reaction, absorbance was measured at 540 nm. L NMMA was used as the positive control. The formula (NO inhibition rate = [(OD_LPS_ − OD_Con_) − (OD_compound_ − OD_Con_)]/(OD_LPS_ − OD_Con_) × 100%) was used to calculate the anti-inflammatory activities.^28^

### Western blotting

RAW 264.7 cells were seeded into 6 well plates at a density of 8 × 10^5^ cells per well. Based on the CCK 8 and NO assay results, three drug concentrations (20, 10, and 5 µM) were selected. Total cellular protein was extracted using 200 µL of freshly prepared RIPA lysis buffer (PMSF: phosphatase inhibitor cocktail: RIPA = 1 : 2 : 100) with gentle shaking. Protein samples were separated by 10% SDS PAGE and transferred onto a PVDF membrane by electroblotting. The membrane was blocked with rapid blocking buffer for 15 min and then incubated with primary antibodies overnight at 4 °C. After washing three times with TBST, the membrane was incubated with HRP conjugated secondary antibodies for 1 h at room temperature. Protein bands were visualized using ECL substrate with 1 min exposure, and images were acquired using a gel imaging system.^29^

### Statistical analysis

One-way analysis of variance (ANOVA) was used to compare differences in cell viability between the compound-treated groups and the normal control group. A four-parameter logistic nonlinear regression model was applied to analyze the dose–response relationship and calculate the half-maximal inhibitory concentration (IC_50_) with 95% confidence intervals. Statistical analysis was performed using GraphPad Prism 10.4.1. All data were presented as the mean ± standard deviation (SD) of at least three independent biological replicates. Statistical significance was defined as *p* < 0.05.

## Conclusions

Five new secondary metabolites, including one diphenolic derivative decemcellulin A (1), one butenolide derivative decemcellulin B (2), two furo-α-pyrone derivatives (±)- decemcellulin C (3), one sesquiterpene derivative decemcellulene A (4), were isolated from the mangrove-derived fungus *Fusarium decemcellulare* DQ-28. Compound 1 exhibited potent anti-inflammatory activity with IC_50_ value of 19.61 ± 0.14 µM. Western blot analysis revealed that 1 selectively downregulated iNOS protein expression induced by LPS in RAW264.7 cells. Compound 1 exhibits a unique C6–C5–C6 carbon skeleton and belongs to diphenolic derivatives. Diphenolics were reported to have anti-inflammatory,^30^ antimicrobial,^31^ cytotoxic,^30^ immunosuppressive^32^ and PTP1B inhibitory^33^ activity.

## Author contributions

Ruxue Mu: data analysis, activity test and writing organic manuscript. Junhao Zhu: strain fermentation cultivation and isolation identification. Zhongqian Xue: data analysis and activity test. Yang Wang: extraction separation. Meng Zhang: data analysis. Xinyao Chu: extraction separation. Jianning Dong: extraction separation. Ge Zou: data analysis, review and editing. Yan Chen: concepts, review and editing, and funding acquisition.

## Conflicts of interest

The authors declare no competing financial interest.

## Supplementary Material

RA-016-D6RA05011E-s001

RA-016-D6RA05011E-s002

RA-016-D6RA05011E-s003

## Data Availability

CCDC 2556838 contains the supplementary crystallographic data for this paper.^34^ Data supporting this study are included within the article and its supplementary information (SI). Supplementary information is available. See DOI: https://doi.org/10.1039/d6ra05011e.
