# Supplementary material for: Metabolites with anti-inflammatory activity from the mangrove endophytic fungus Fusarium decemcellulare DQ-28
Source: RSC Adv. 2026 Jul 13;16(37):39926–32. doi: 10.1039/d6ra05011e (PMC13358892; doi:10.1039/d6ra05011e)
Supplement: Supplementary file 1 [file RA-016-D6RA05011E-s001.pdf]

Supplementary Information (SI) for RSC Advances.
This journal is © The Royal Society of Chemistry 2026

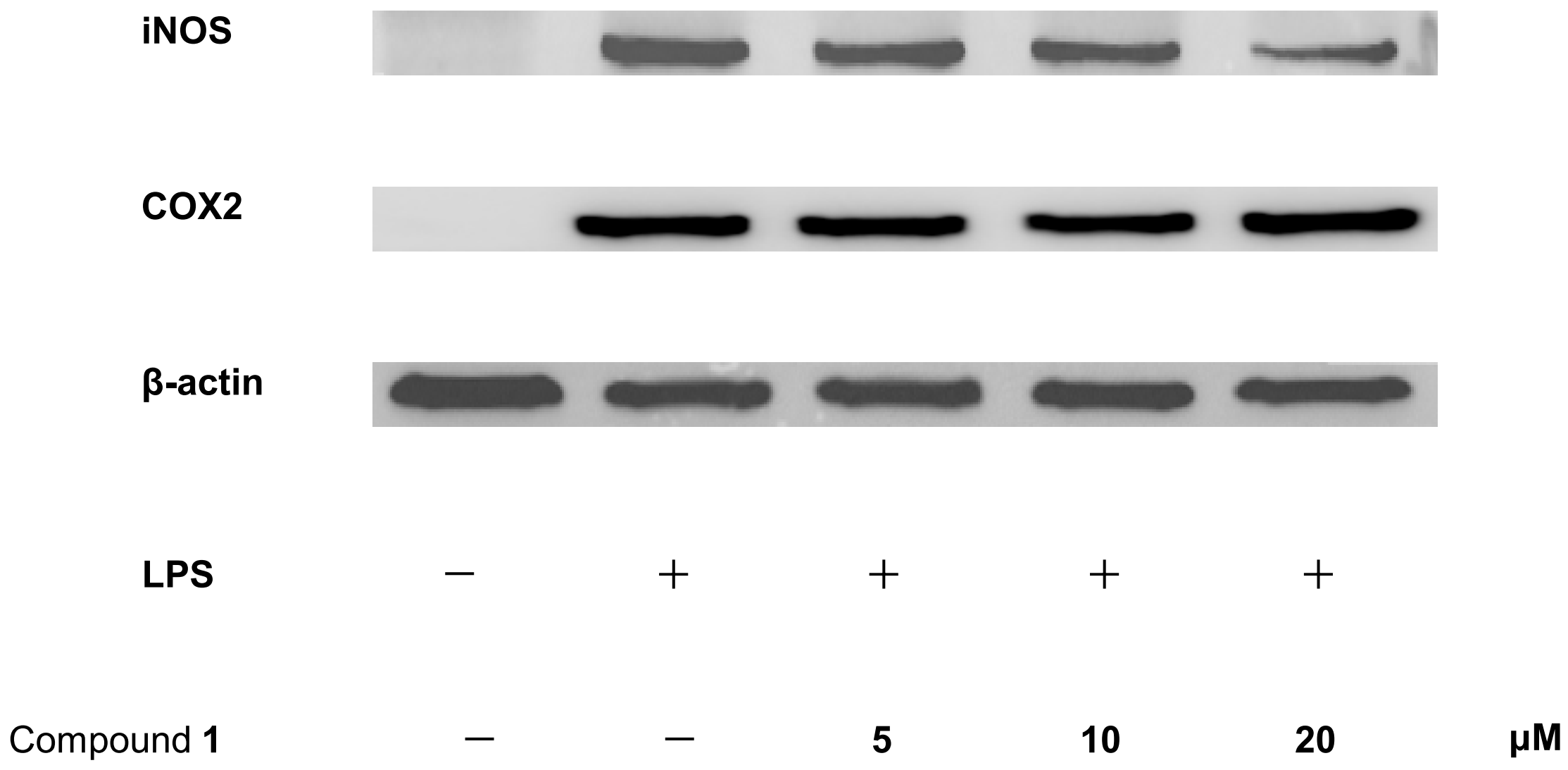

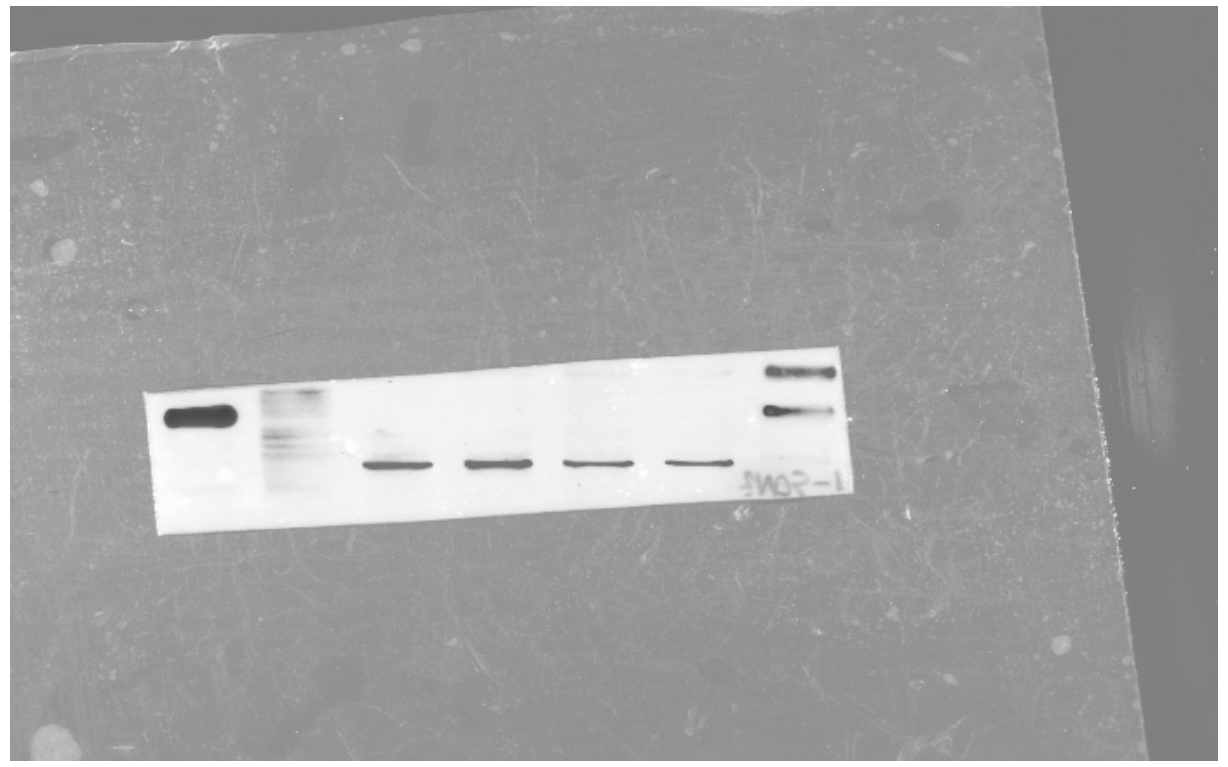

iNOS-1

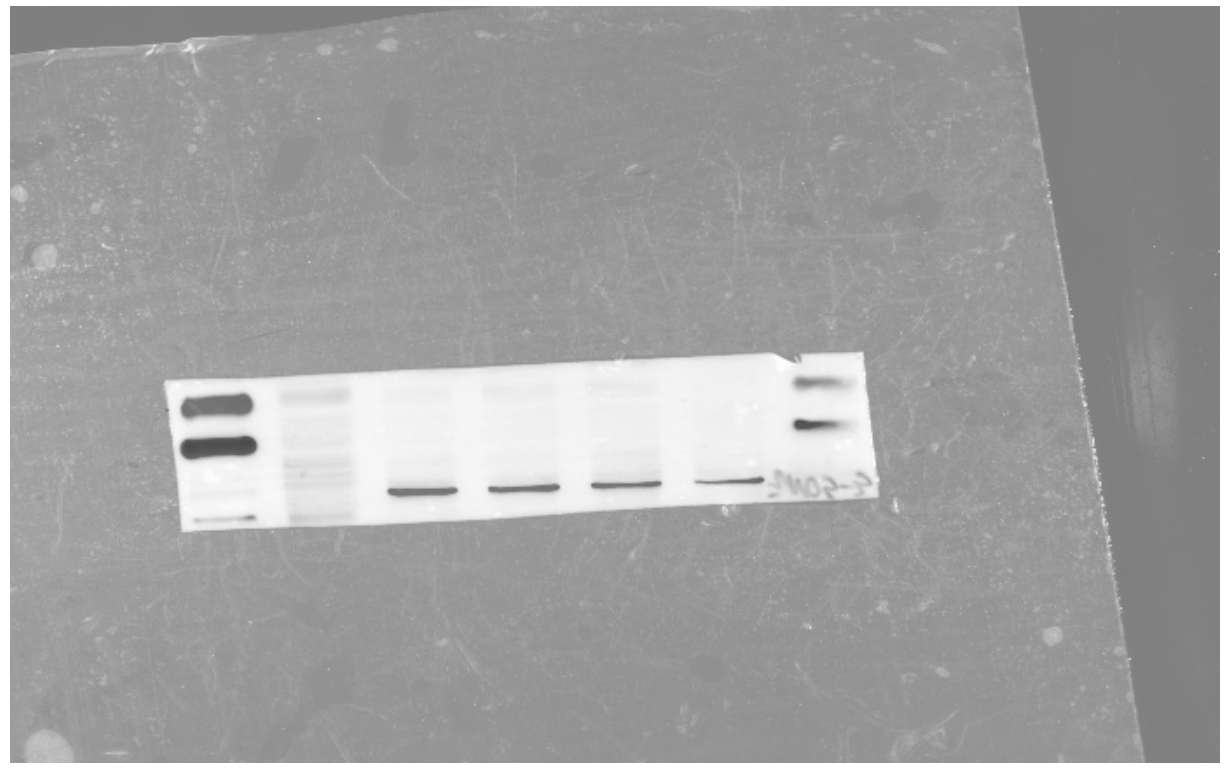

iNOS-2

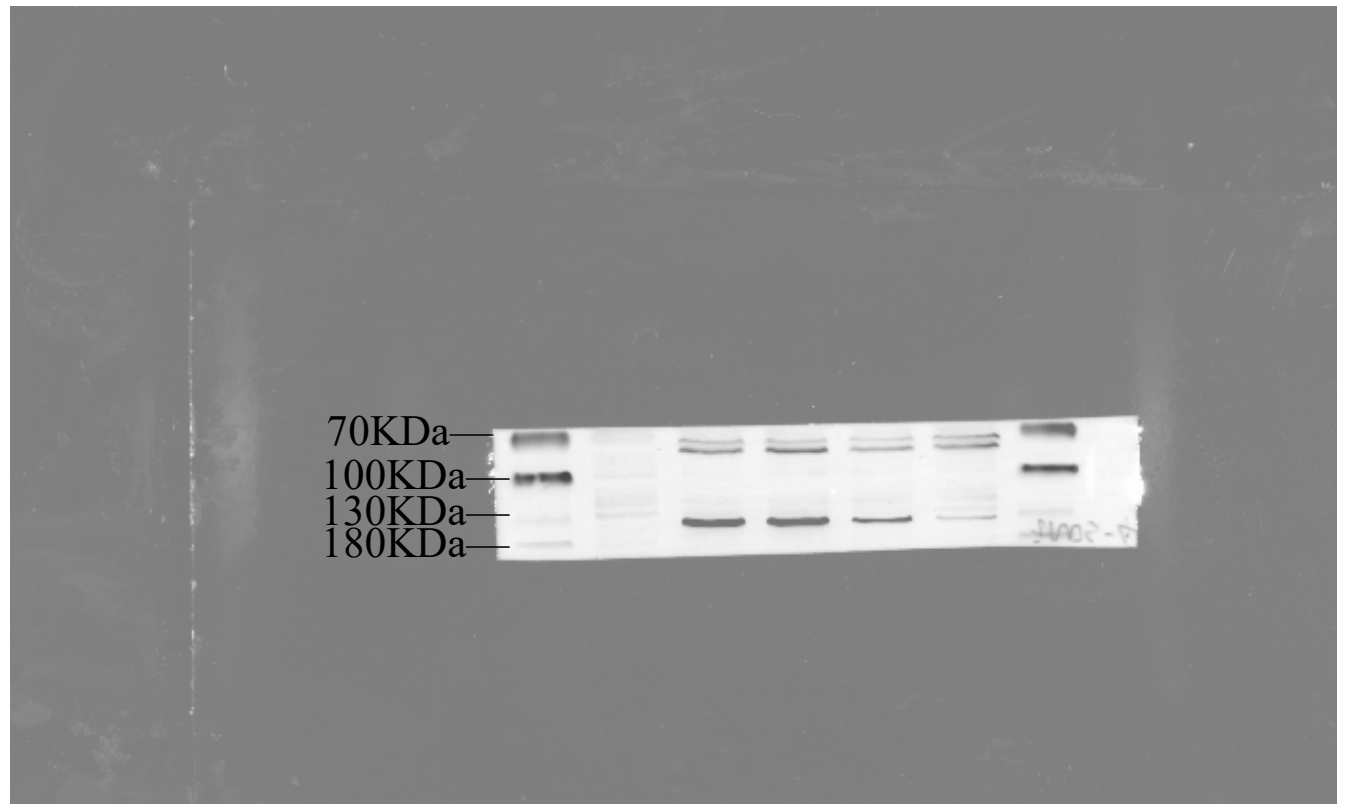

iNOS-3

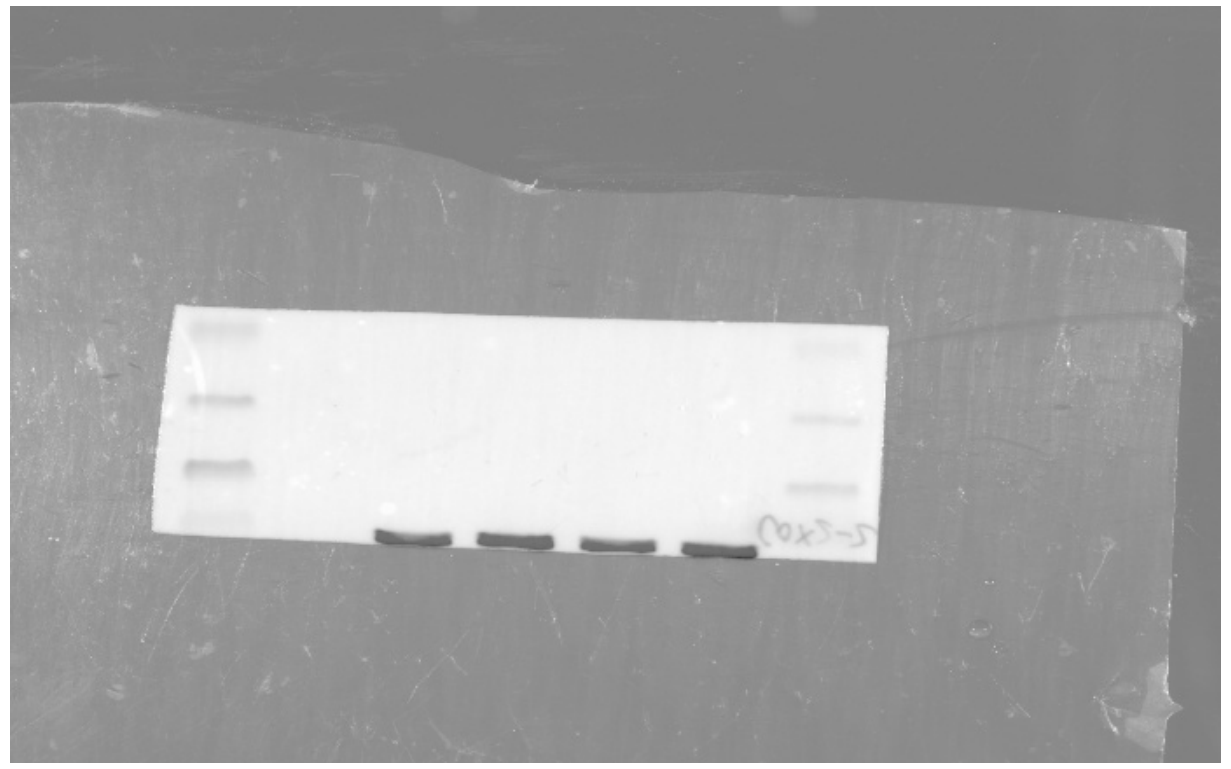

COX-2-1

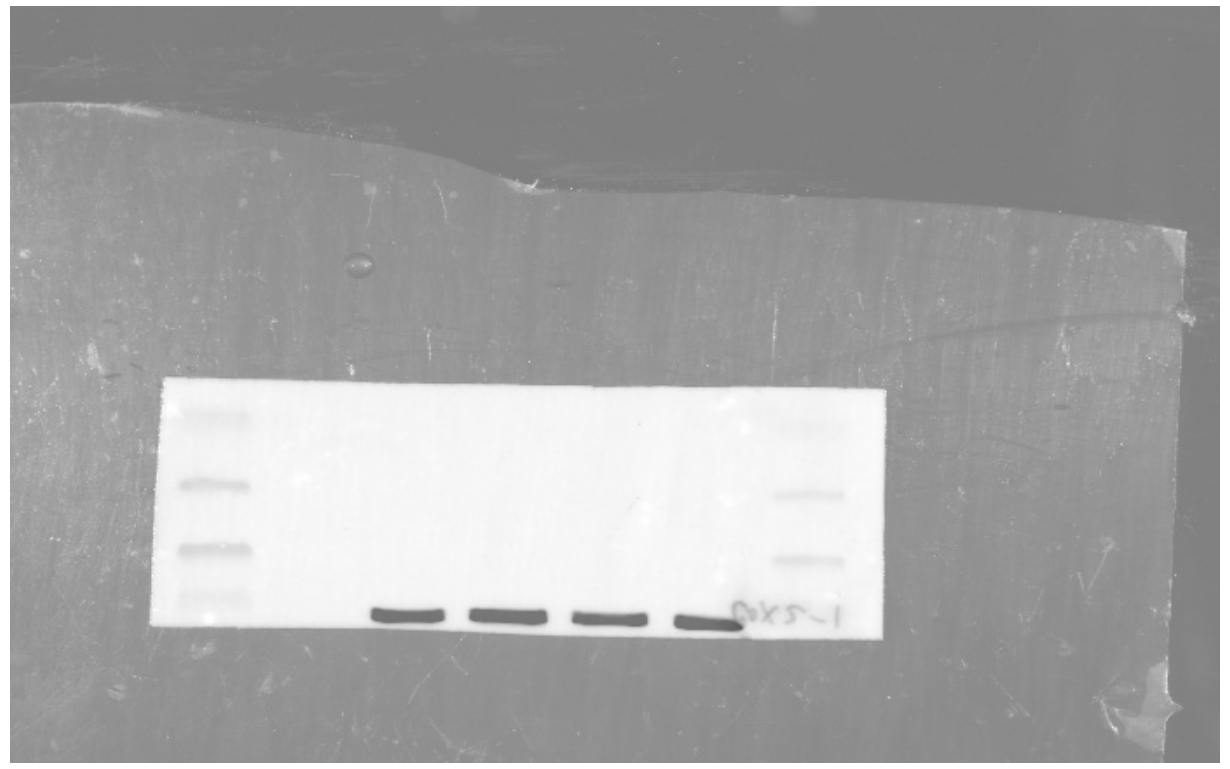

COX-2-1

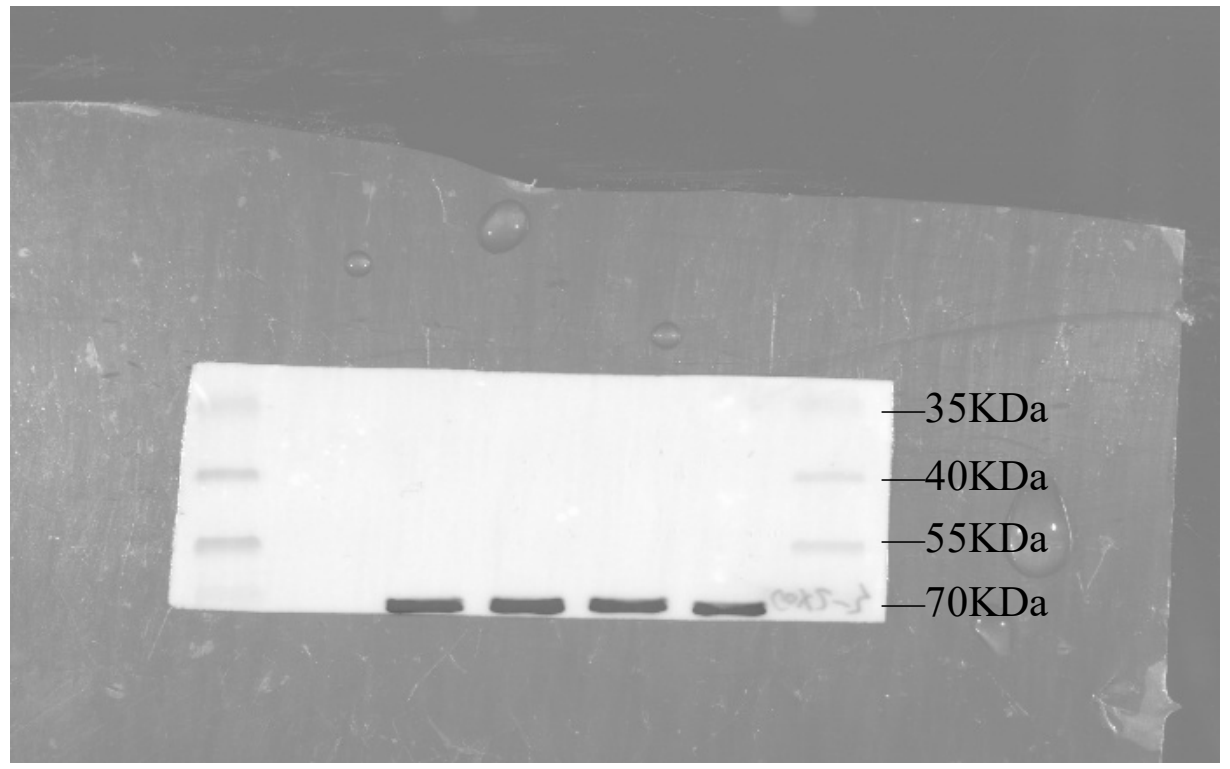

COX-2-3

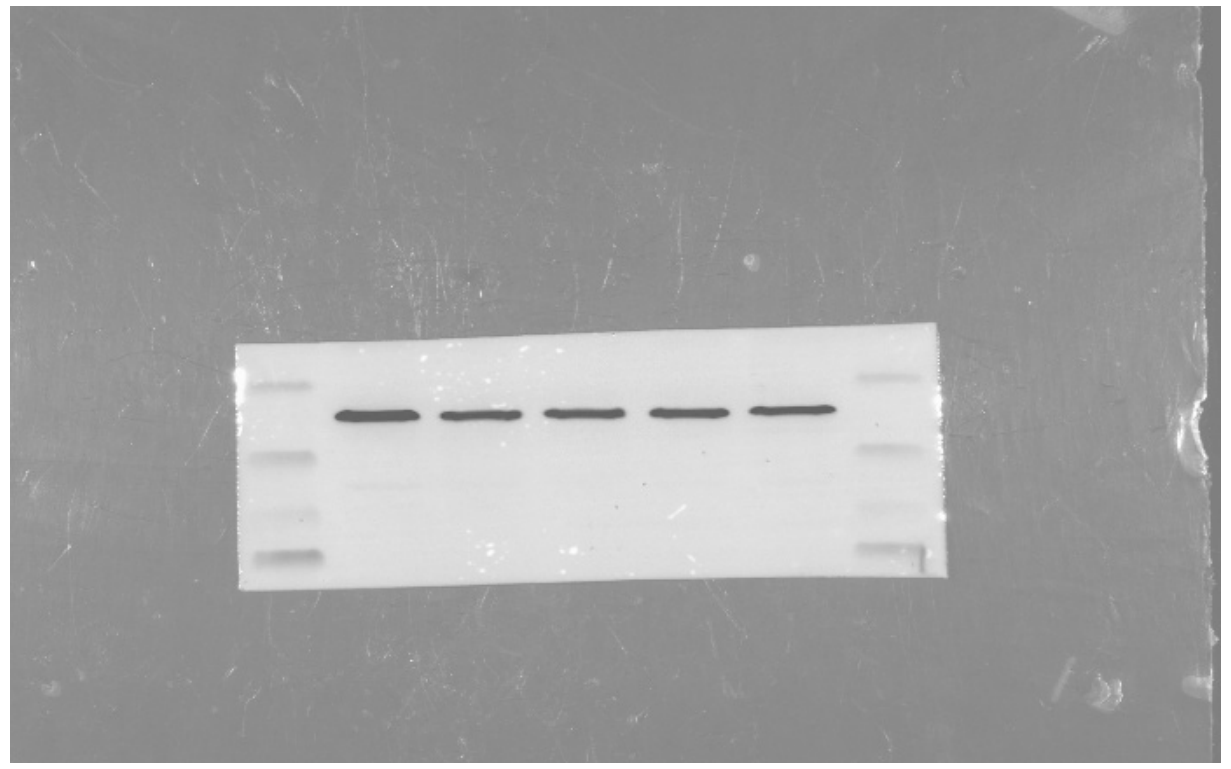

β-actin-1

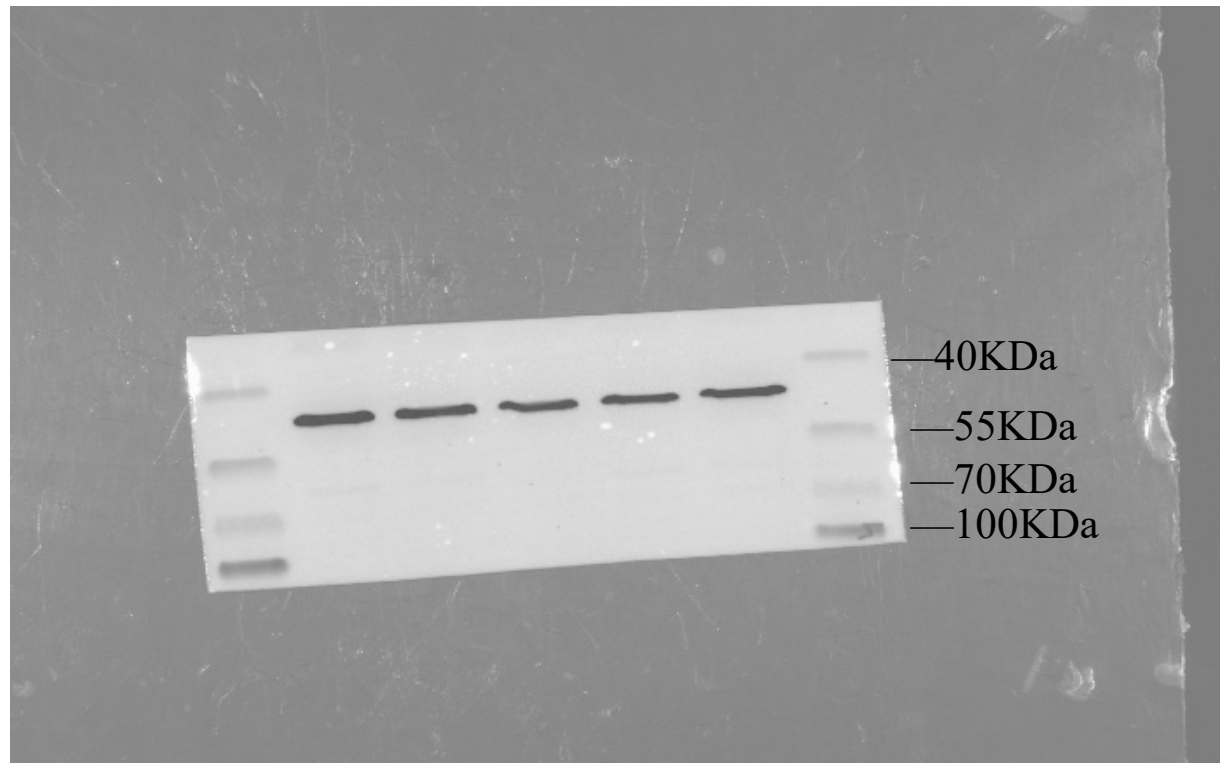

β-actin-2

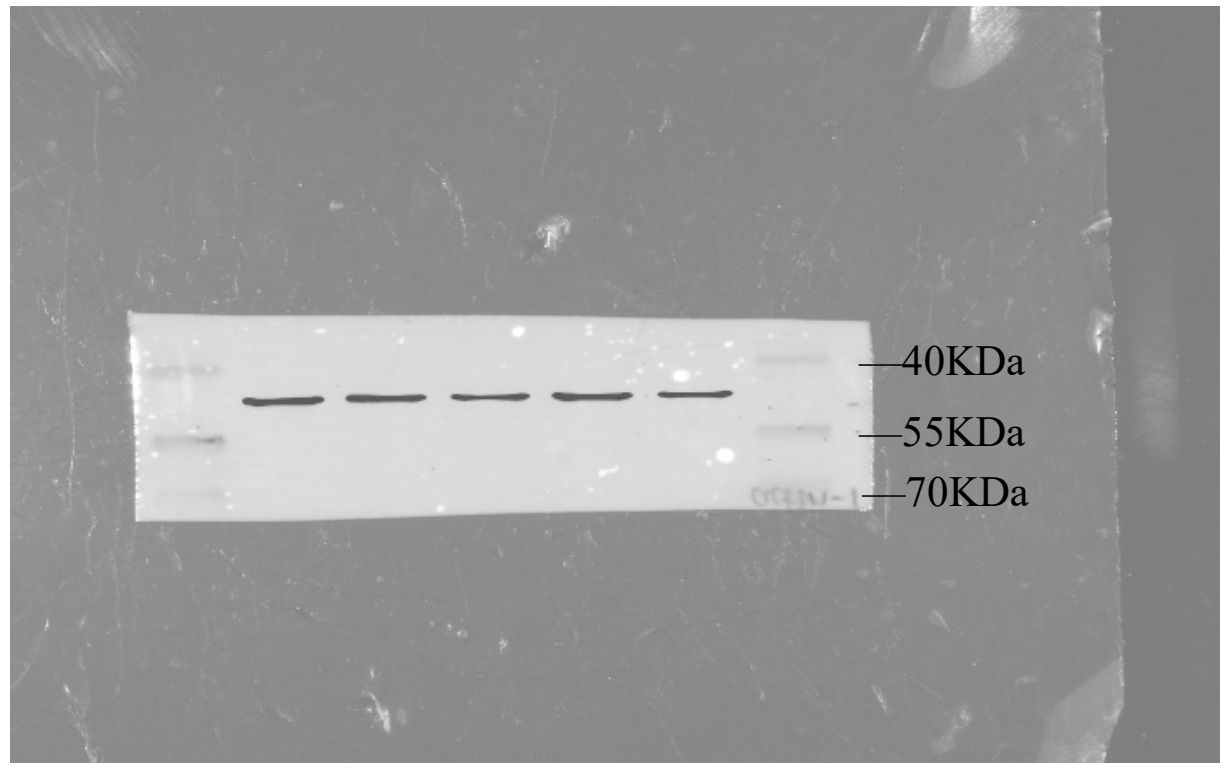

β-actin-3
