# Supplementary material for: Metabolites with anti-inflammatory activity from the mangrove endophytic fungus Fusarium decemcellulare DQ-28
Source: RSC Adv. 2026 Jul 13;16(37):39926–32. doi: 10.1039/d6ra05011e (PMC13358892; doi:10.1039/d6ra05011e)
Supplement: Supplementary file 2 [file RA-016-D6RA05011E-s002.pdf]

Supplementary Information (SI) for RSC Advances.
This journal is © The Royal Society of Chemistry 2026

Supplementary Material

Metabolites with Anti-inflammatory activity from the Mangrove Endophytic Fungus *Fusarium decemcellulare* DQ-28

Ruxue Mu[a,†], Junhao Zhu[a,†], zhongqian Xue[a], Yang Wang[a], Meng Zhang[a], Xinyao Chu[a], Jianning Dong[a], Ge Zou[b,*], Yan Chen[a,c,*]

[a]School of pharmacy, Anhui Medical University, Hefei, China;

[b]School of Pharmacy, Xianning Medical College, Hubei University of Science and Technology, Xianning, China.

[c]The First Affiliated Hospital of Anhui Medical University, Hefei, China;

*Correspondence author: cychemistry@ahmu.edu.cn (Y. Chen)

[†]Ruxue Mu and Junhao Zhu contributed equally.

Supporting Information Contents:

Figure. S1 ^{1}H NMR spectrum of compound **1** (500 MHz, $CDCl_3$).
Figure. S2 ^{13}C NMR spectrum of compound **1** (125 MHz, $CDCl_3$).
Figure. S3 HSQC spectrum of compound **1**.
Figure. S4 HMBC spectrum of compound **1**.
Figure. S5 NOESY spectrum of compound **1**.
Figure. S6 HRESIMS spectrum of compound **1**.
Figure. S7 ^{1}H NMR spectrum of compound **2** (500 MHz, MeOD-d_4).
Figure. S8 ^{13}C NMR spectrum of compound **2** (125 MHz, MeOD-d_4).
Figure. S9 HSQC spectrum of compound **2**.
Figure. S10 ^{1}H-^{1}H COSY spectrum of compound **2**.
Figure. S11 HMBC spectrum of compound **2**.
Figure. S12 HRESIMS spectrum of compound **2**.
Figure. S13 ^{1}H NMR spectrum of compound **3** (500 MHz, $CDCl_3$).
Figure. S14 ^{13}C NMR spectrum of compound **3** (125 MHz, $CDCl_3$).
Figure. S15 HSQC spectrum of compound **3**.
Figure. S16 ^{1}H-^{1}H COSY spectrum of compound **3**.
Figure. S17 HMBC spectrum of compound **3**.
Figure. S18 NOESY spectrum of compound **1**.
Figure. S19 HRESIMS spectrum of compound **3**.
Figure. S20 ^{1}H NMR spectrum of compound **4** (500 MHz, $CDCl_3$).
Figure. S21 ^{13}C NMR spectrum of compound **4** (125 MHz, $CDCl_3$).
Figure. S22 HSQC spectrum of compound **4**.
Figure. S23 ^{1}H-^{1}H COSY spectrum of compound **4**.
Figure. S24 HMBC spectrum of compound **4**.
Figure. S25 NOESY spectrum of compound **1**.
Figure. S26 HRESIMS spectrum of compound **4**.
Figure S27. Comparison of calculated and experimental ^{13}C NMR data of **1**.
Figure S28. Experimental and calculated ECD spectra of compounds **2-4** in MeOH.
Figure S29. Chromatograms obtained from HPLC analyses of **2**.
Figure S30. Chromatograms obtained from chiral HPLC analyses of **3**.
Table S1. The DP4+ evaluation of decemcellulin A (**1**).
Table S2 ^{1}H NMR (500MHz) and ^{13}C NMR (125MHz) data of compounds **2-3**.
Table S3 ^{1}H NMR (500MHz) and ^{13}C NMR (125MHz) data of compound **4** in $CDCl_3$.

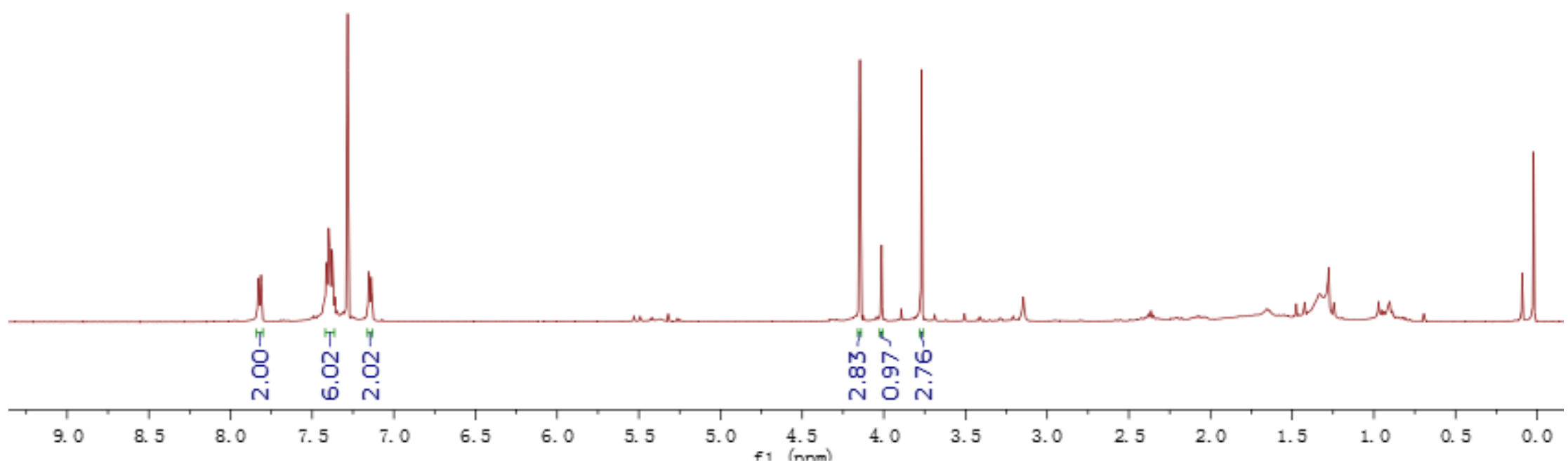

Figure. S1 ^{1}H NMR spectrum of compound **1** (500 MHz, $CDCl_3$).

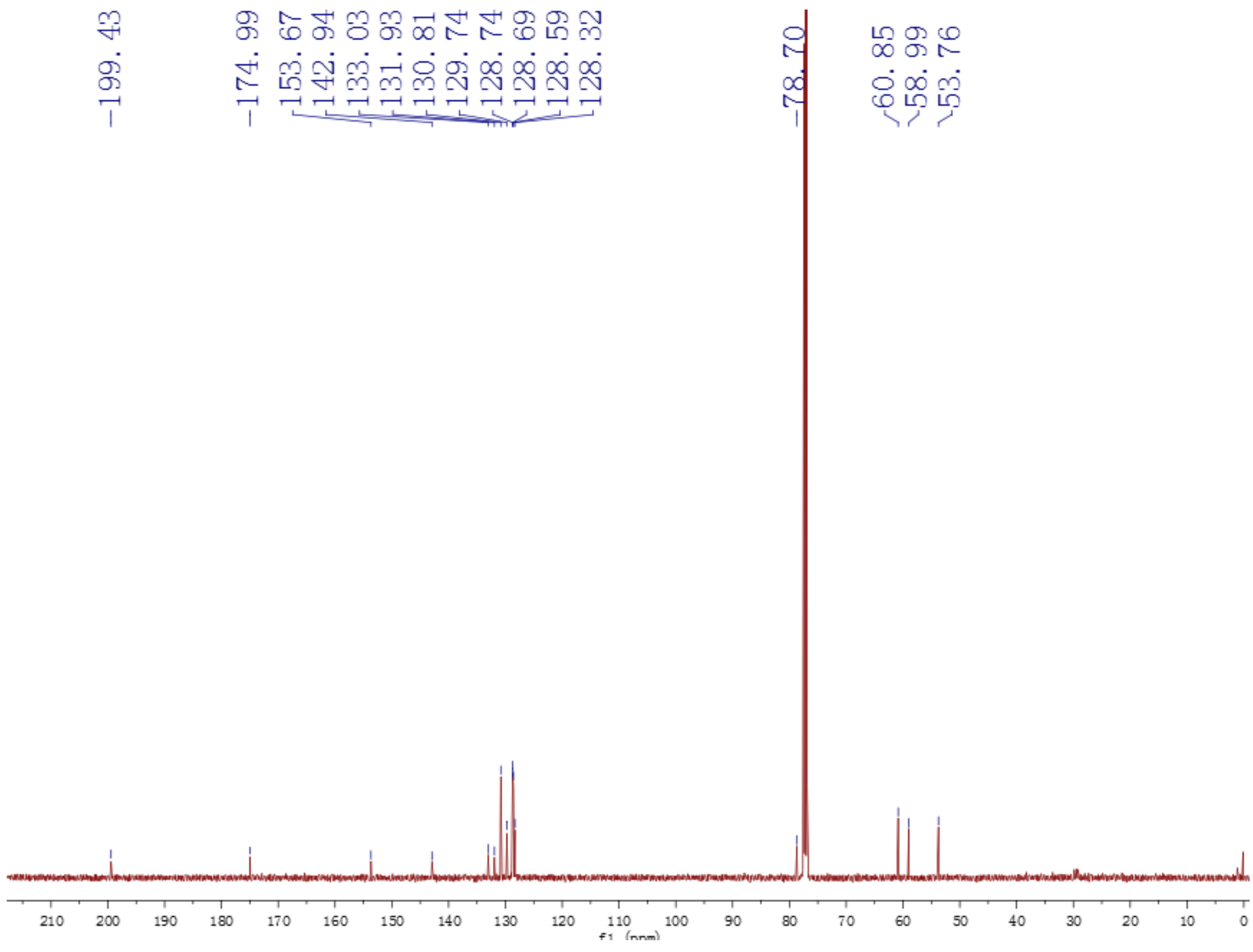

Figure. S2 ^{13}C NMR spectrum of compound **1** (125 MHz, $CDCl_3$).

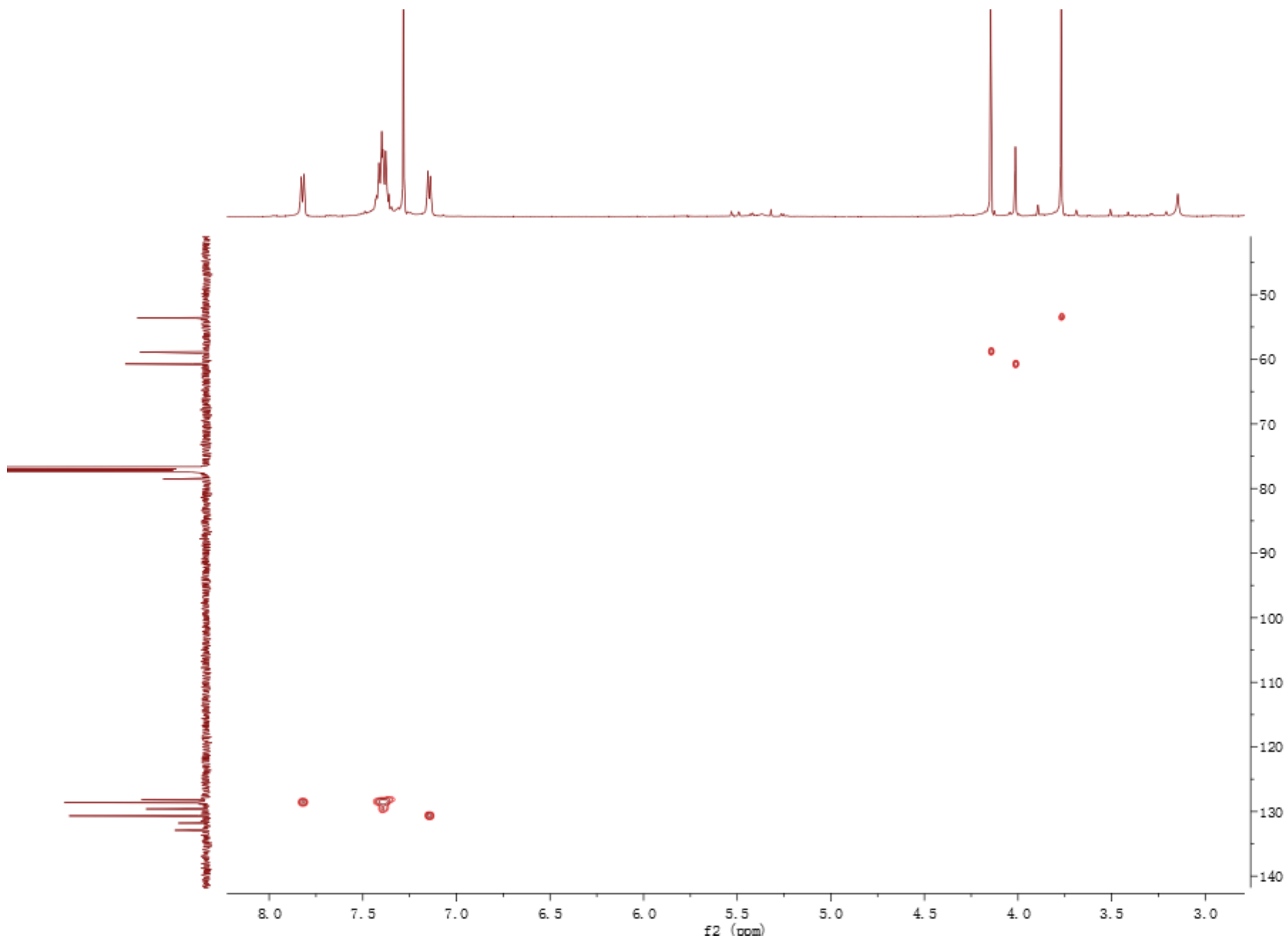

Figure. S3 HSQC spectrum of compound **1**.

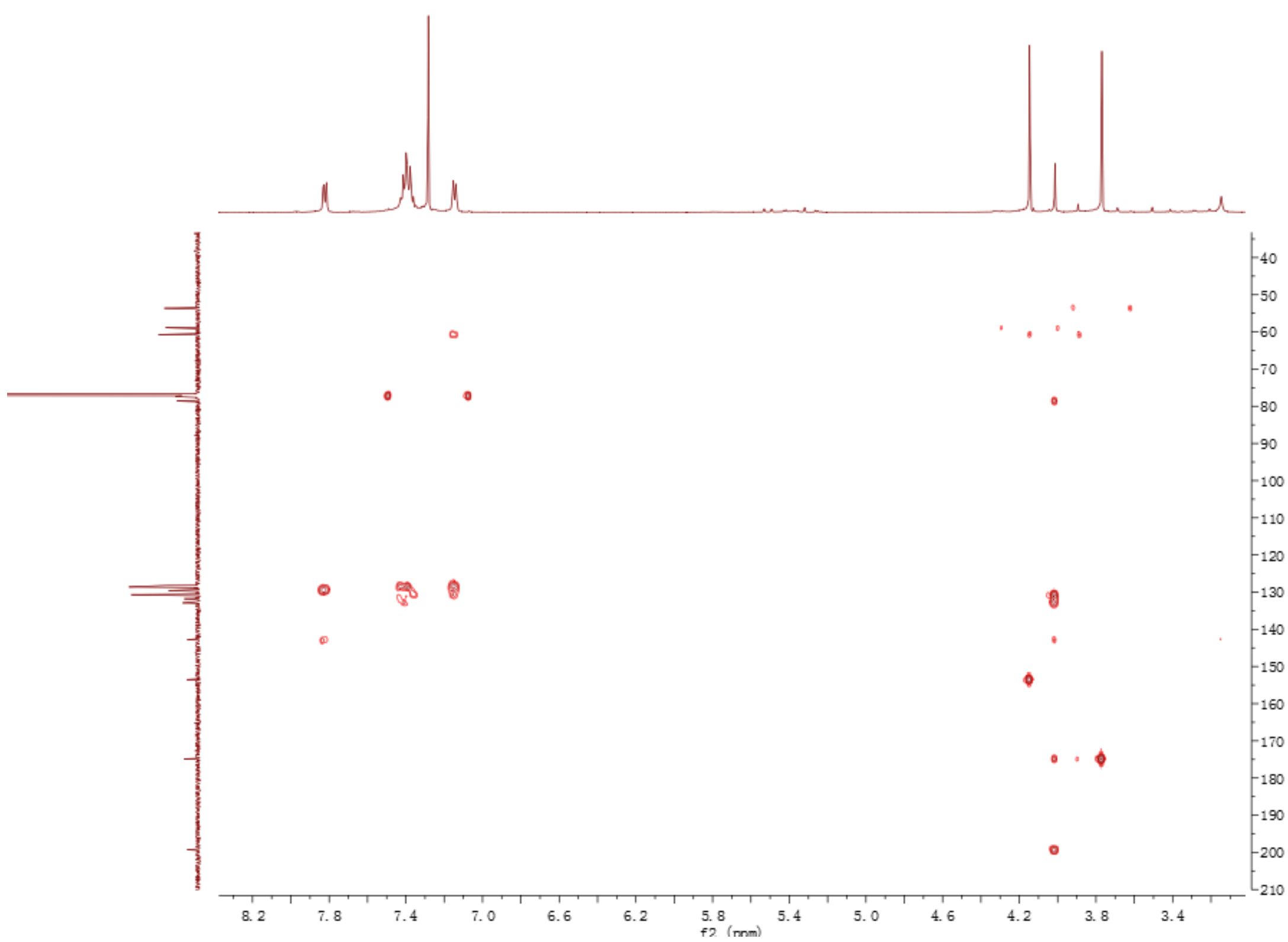

Figure. S4 HMBC spectrum of compound **1**.

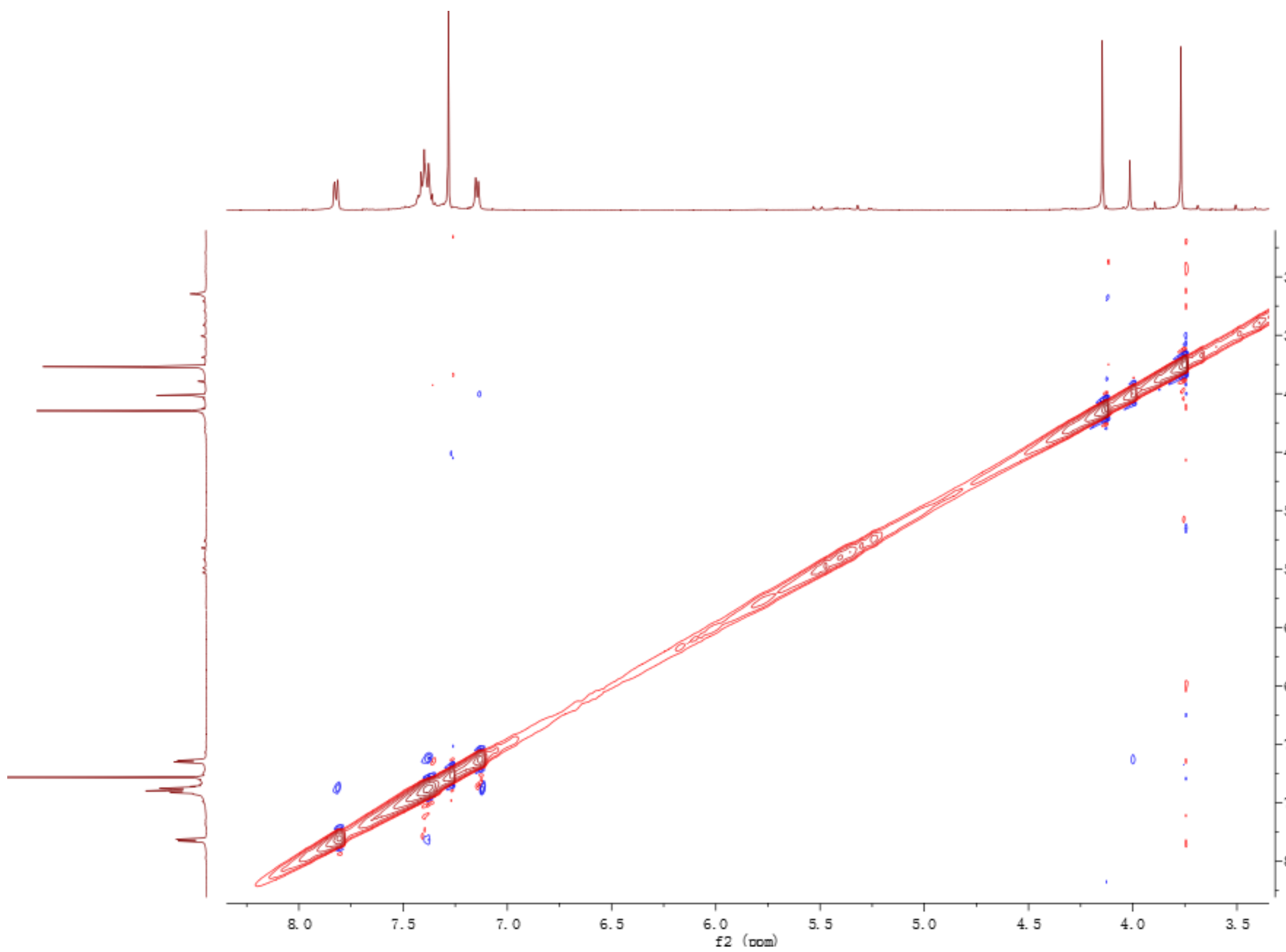

Figure. S5 NOESY spectrum of compound **1**.

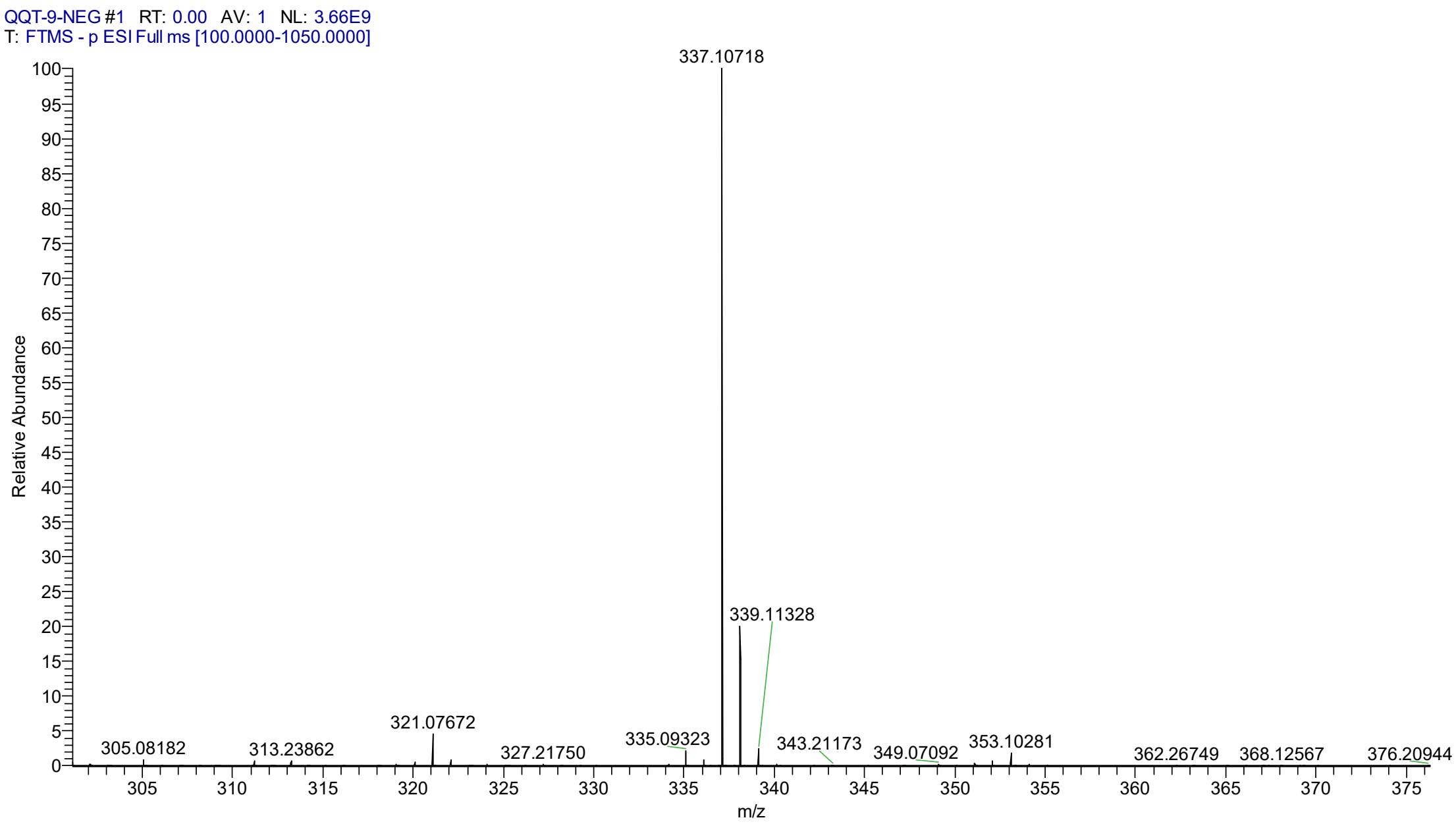

Figure. S6 HRESIMS spectrum of compound **1**.

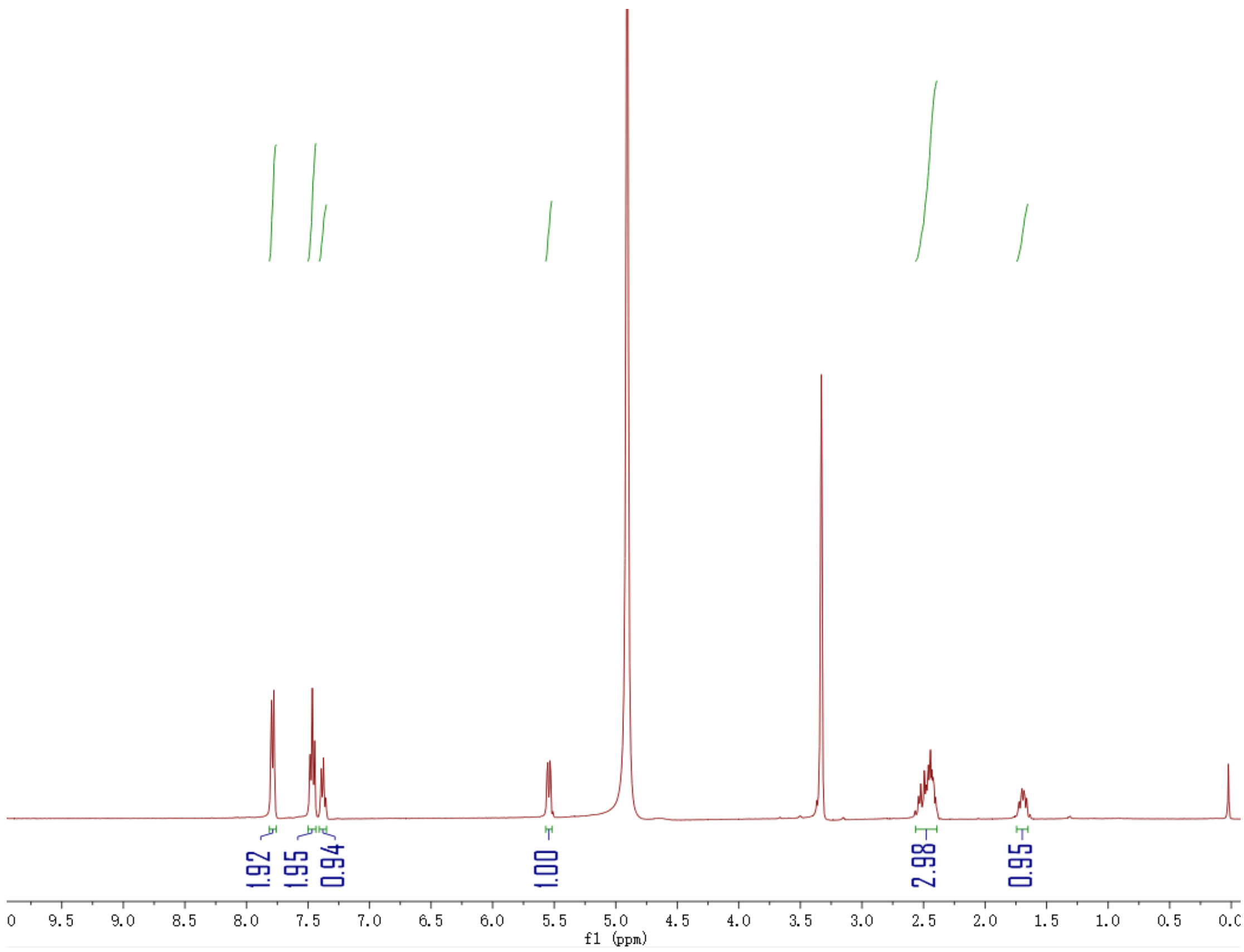

Figure. S7 ^{1}H NMR spectrum of compound **2** (500 MHz, MeOD-d_4).

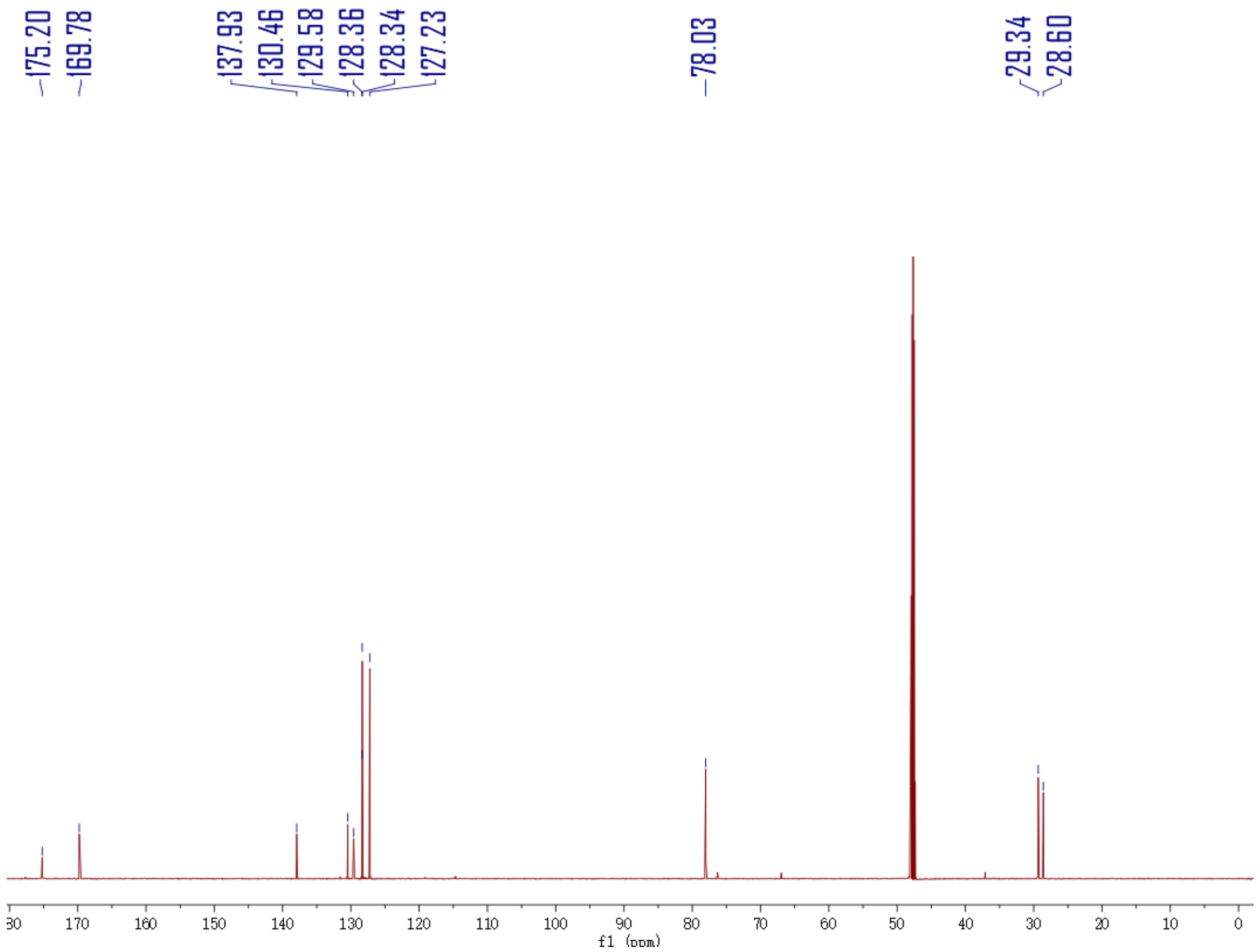

Figure. S8 ^{13}C NMR spectrum of compound **2** (125 MHz, MeOD-d_4).

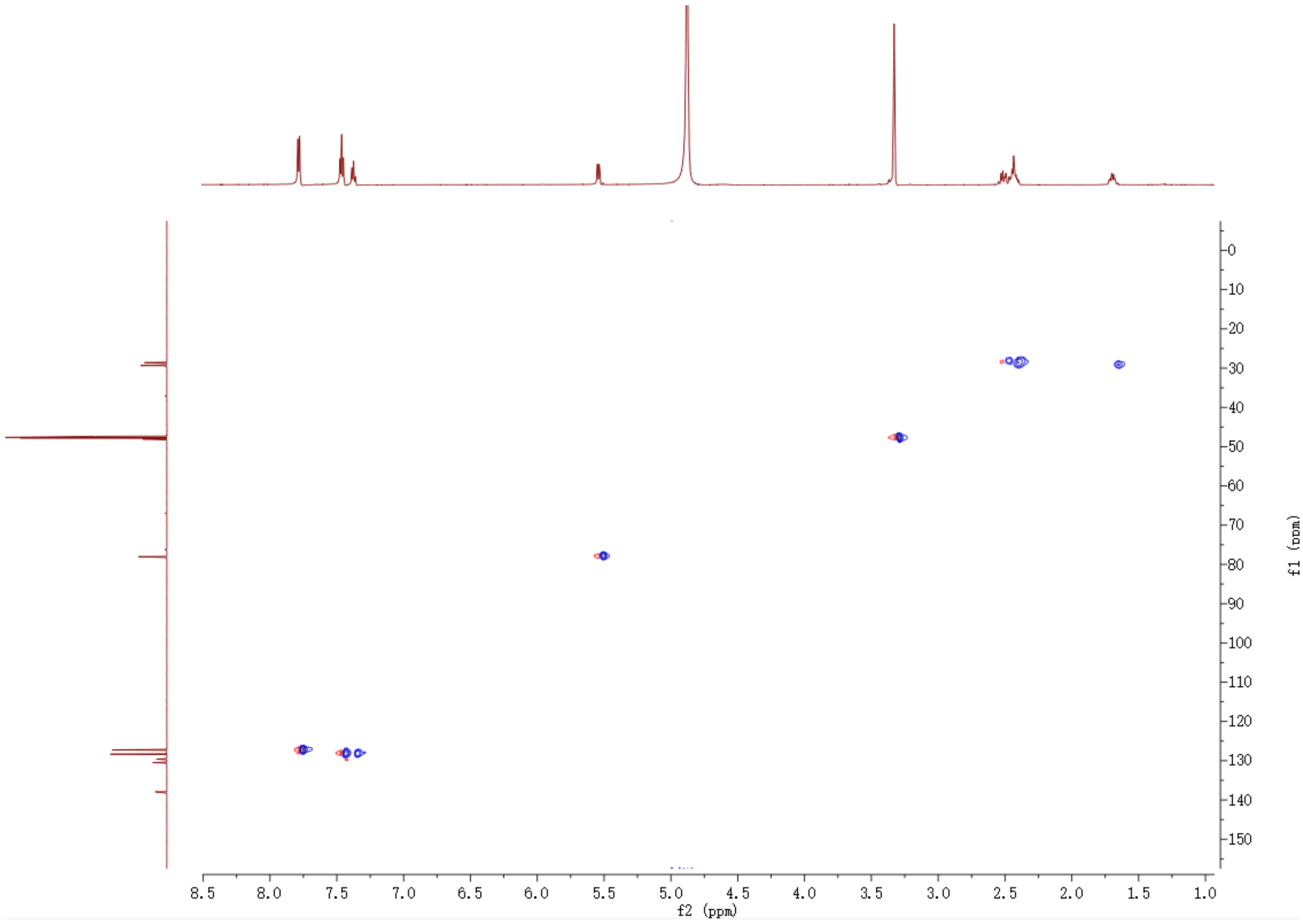

Figure. S9 HSQC spectrum of compound **2**.

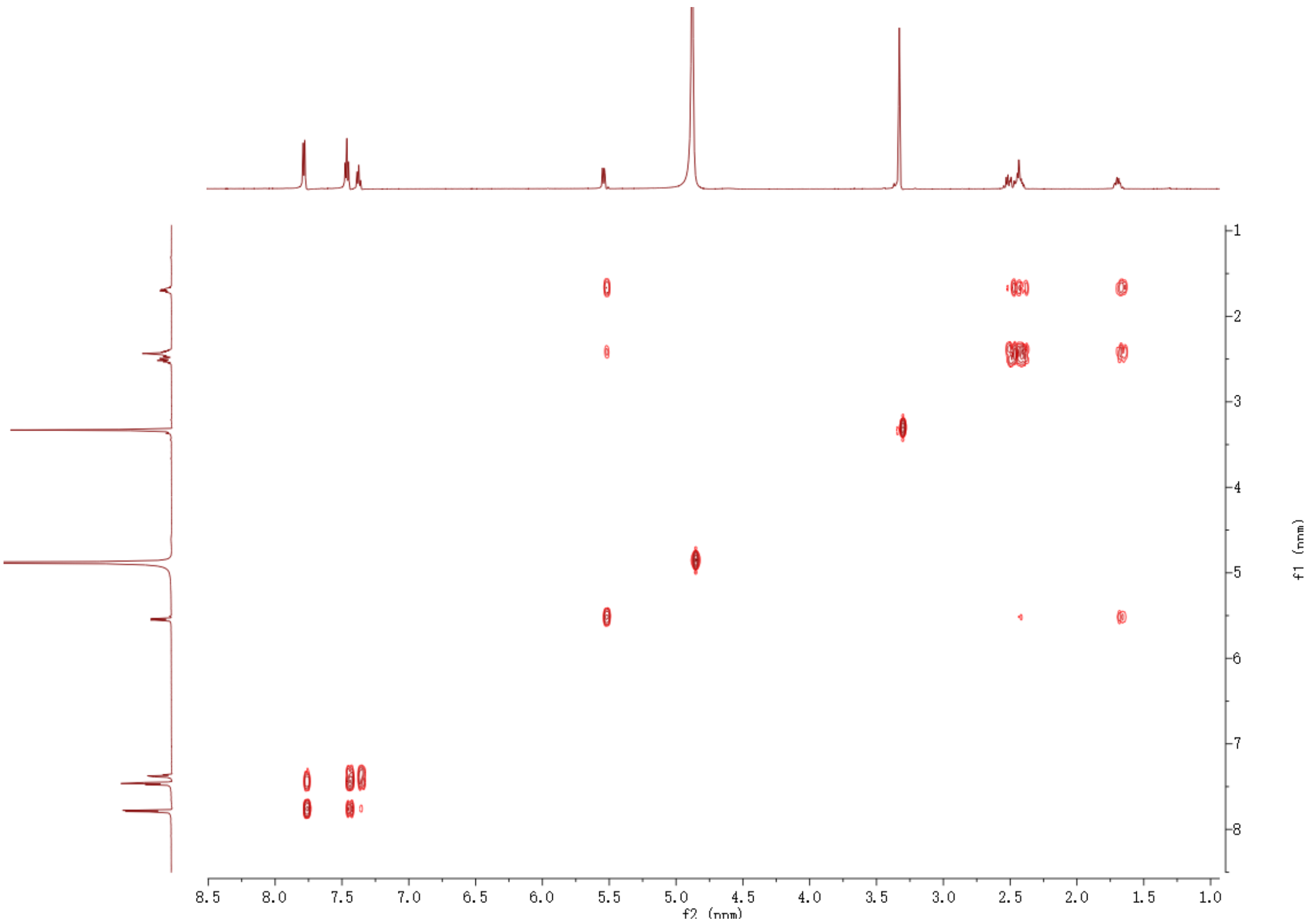

Figure. S10 ^{1}H-^{1}H COSY spectrum of compound **2**.

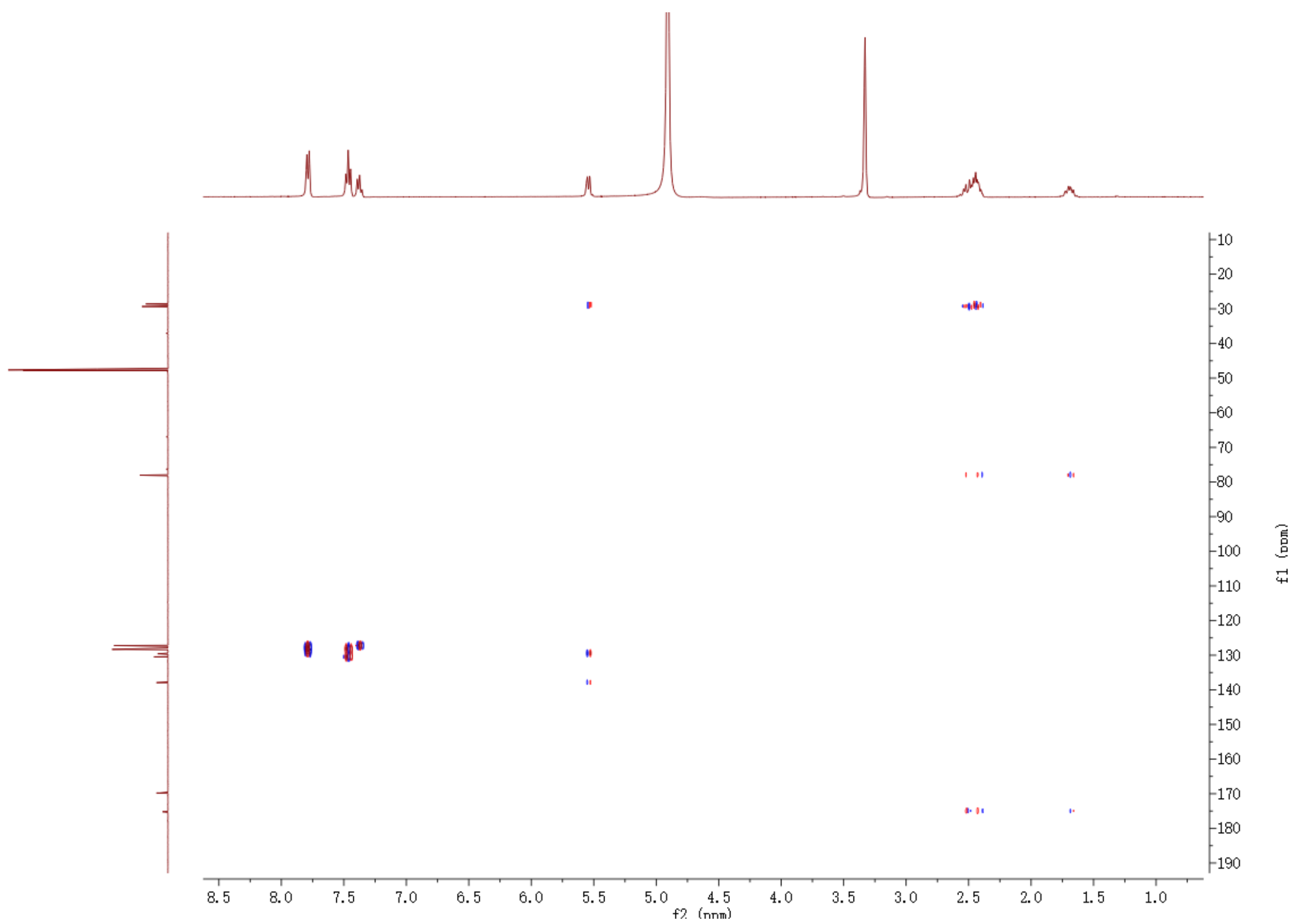

Figure. S11 HMBC spectrum of compound **2**.

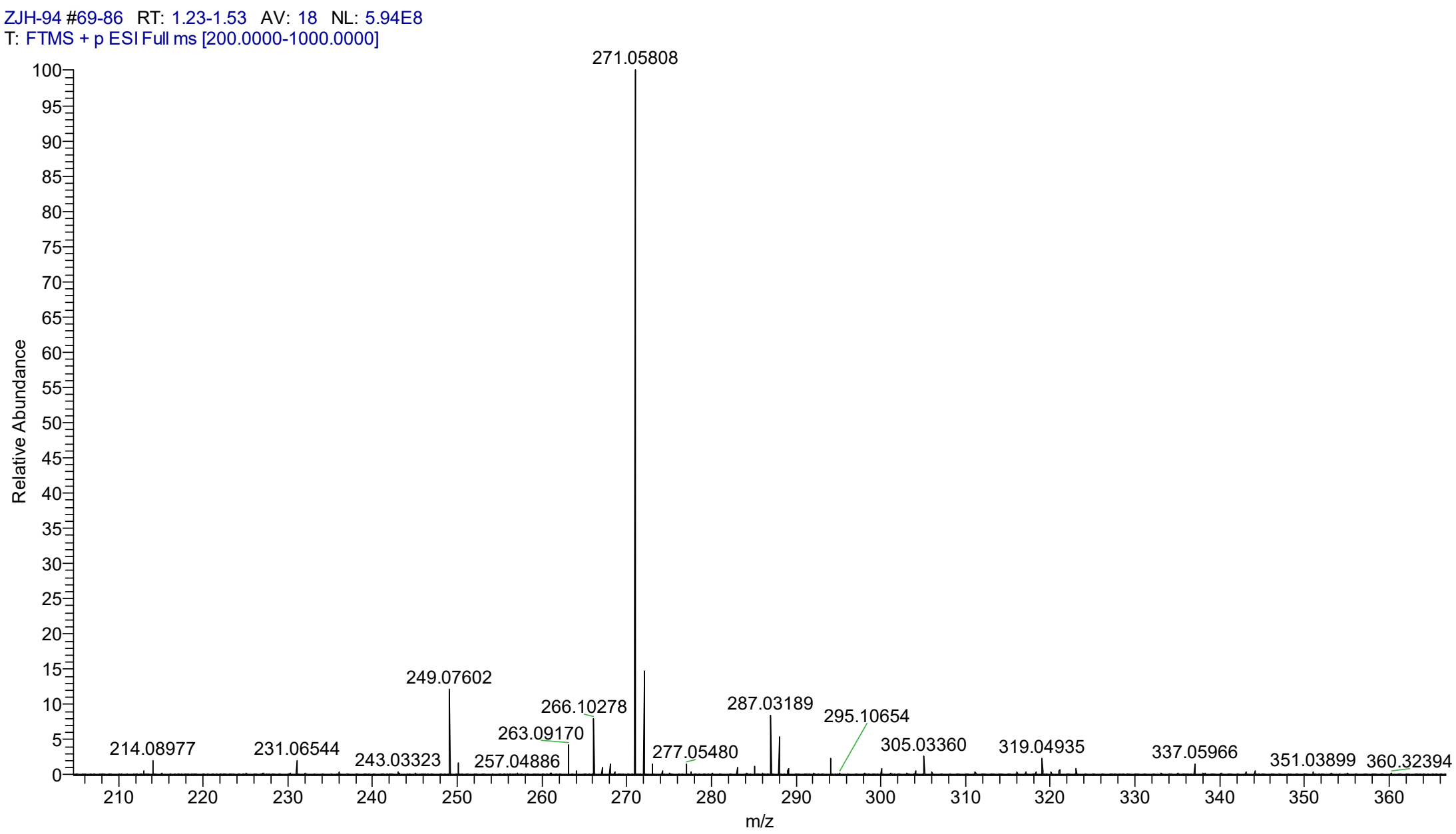

Figure. S12 HRESIMS spectrum of compound **2**.

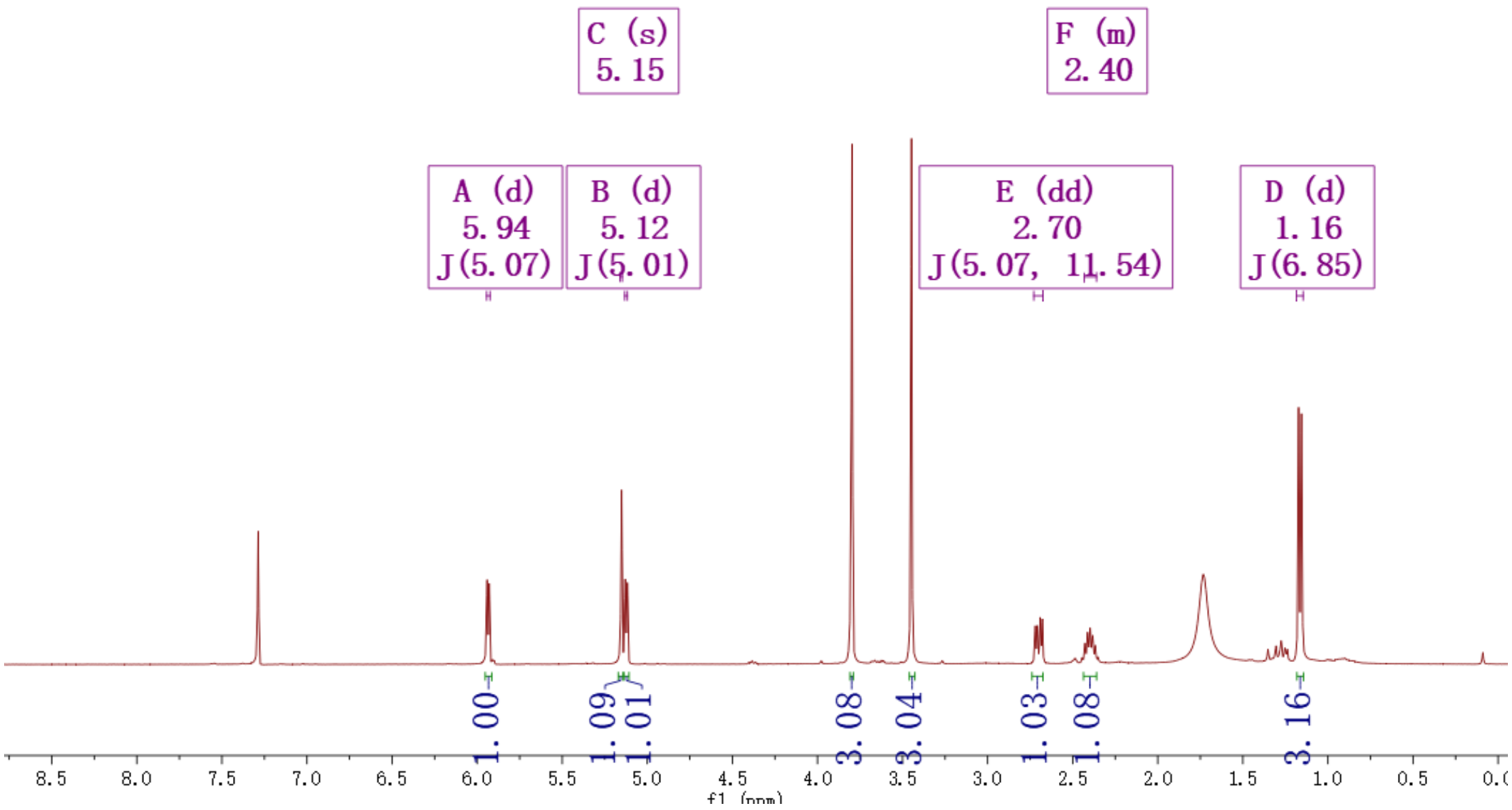

Figure. S13 ^{1}H NMR spectrum of compound **3** (500 MHz, $CDCl_3$).

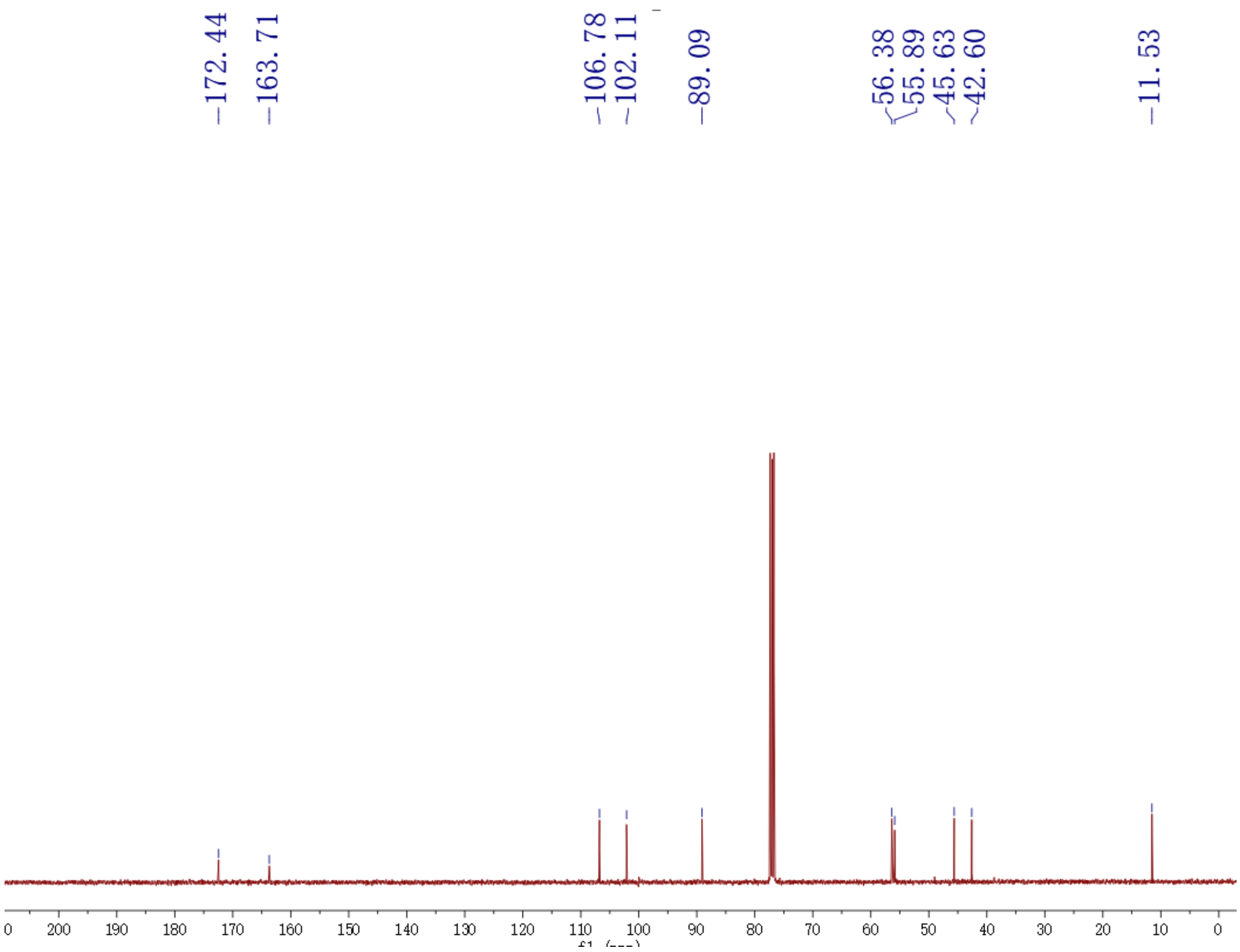

Figure. S14 ^{13}C NMR spectrum of compound **3** (125 MHz, $CDCl_3$).

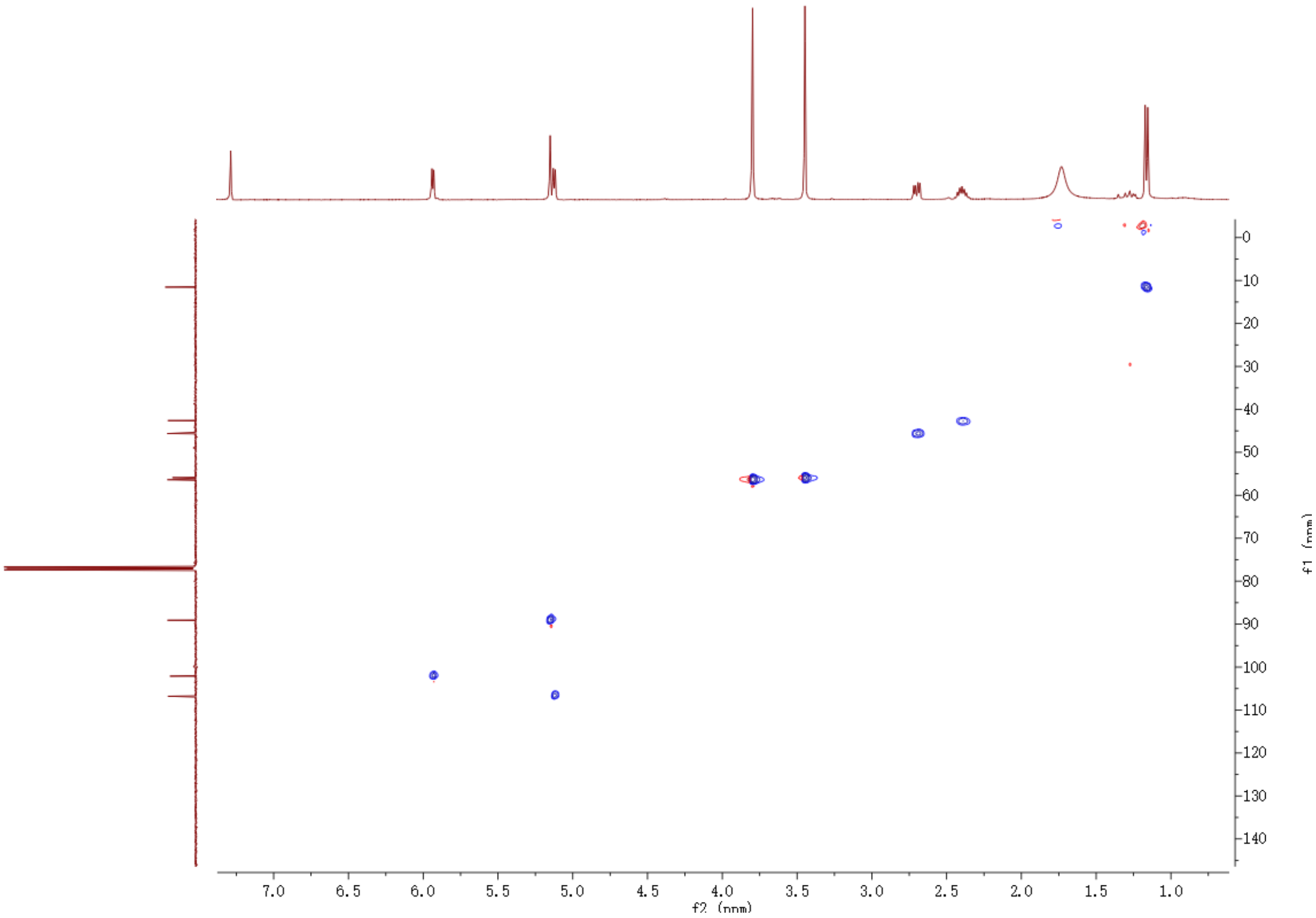

Figure. S15 HSQC spectrum of compound **3**.

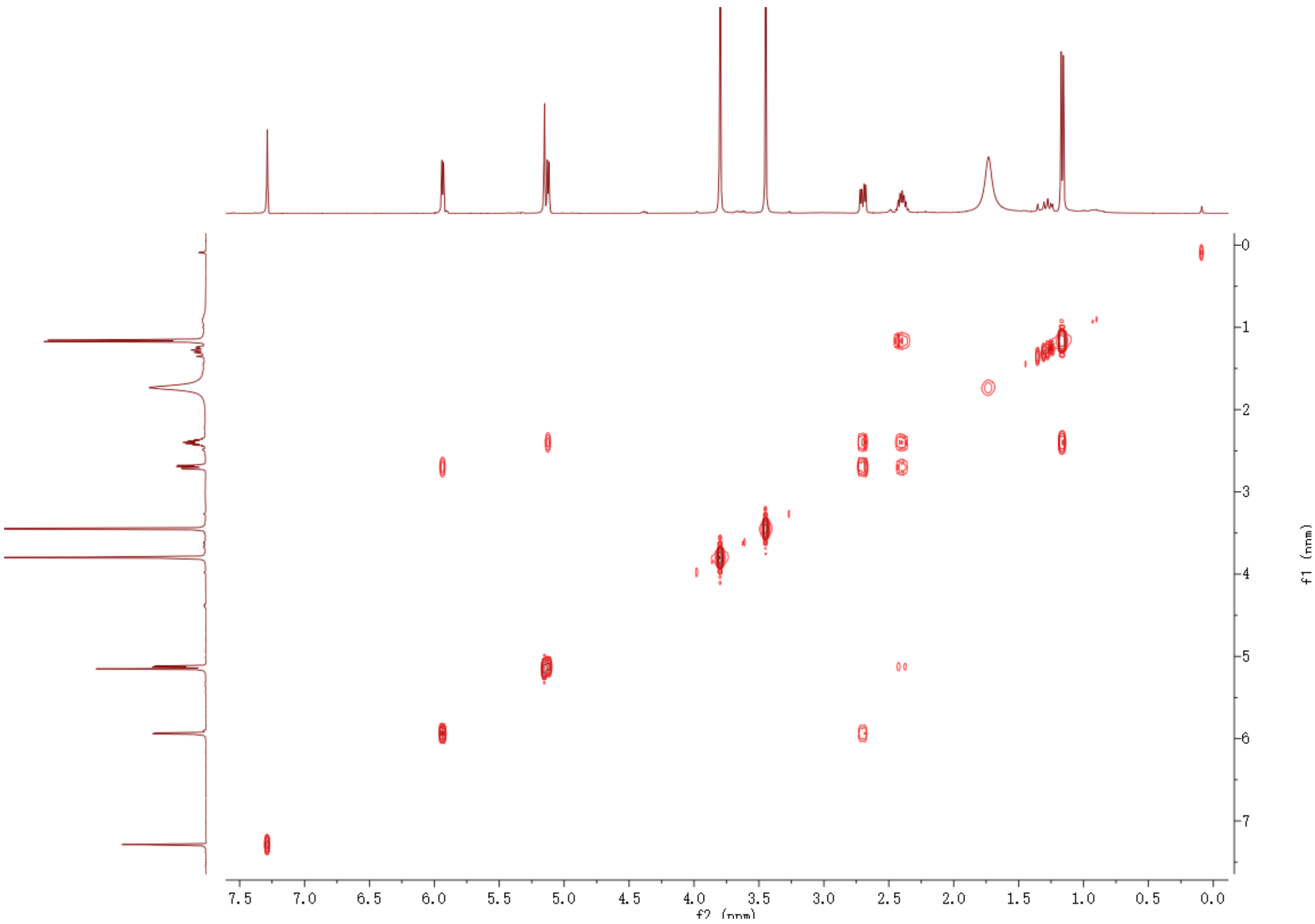

Figure. S16 ^{1}H-^{1}H COSY spectrum of compound **3**.

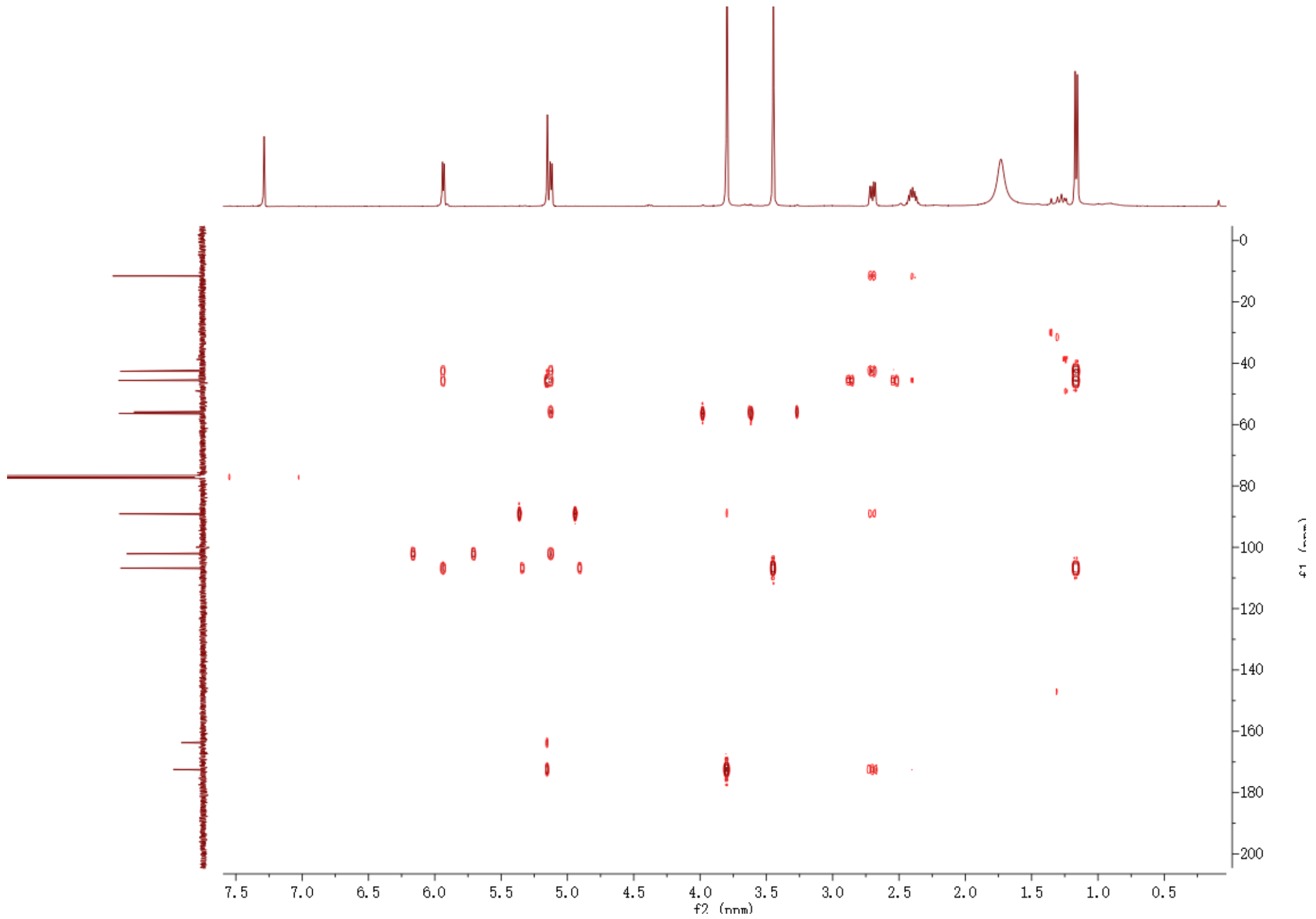

Figure. S17 HMBC spectrum of compound **3**.

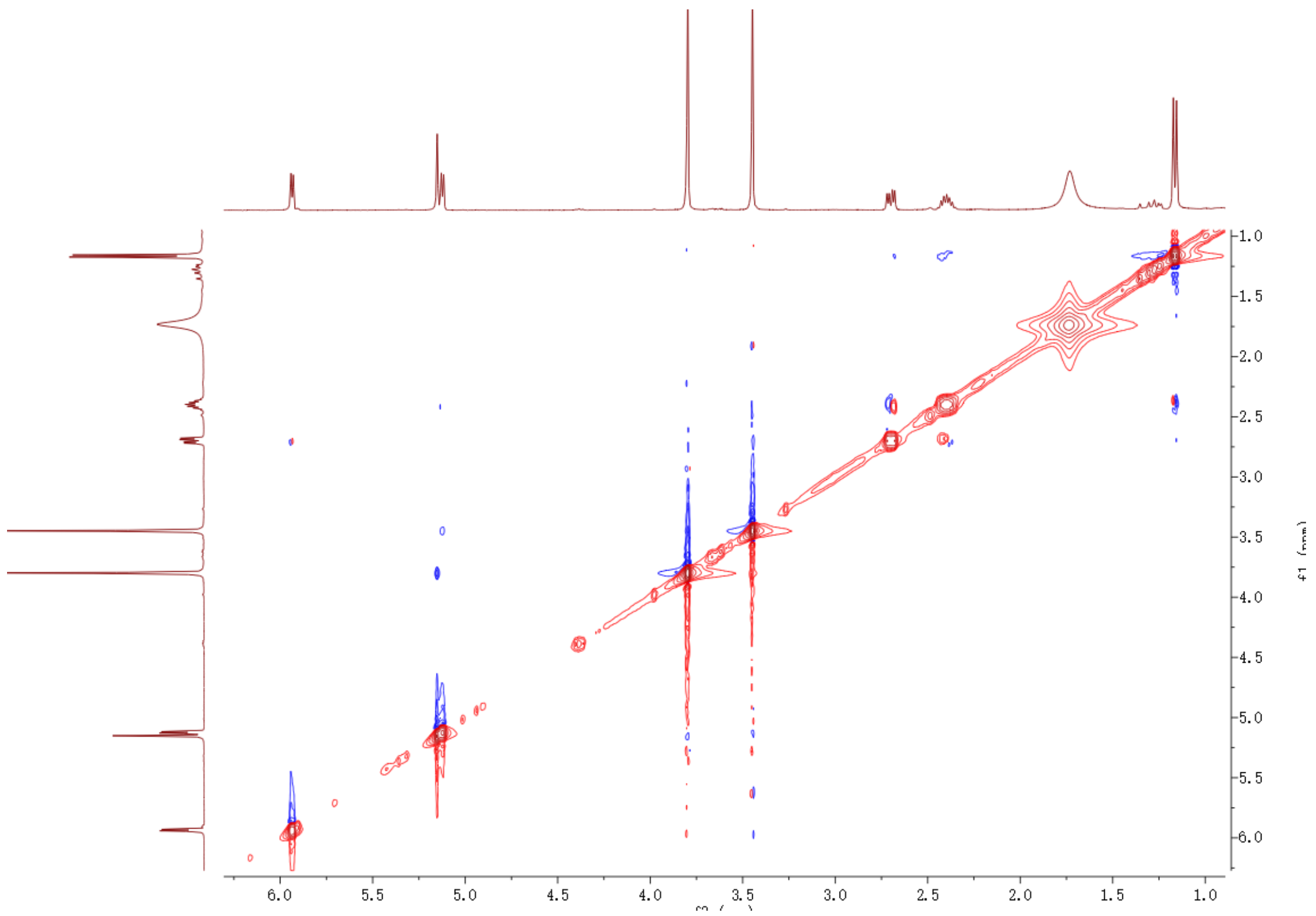

Figure. S18 NOESY spectrum of compound **3**.

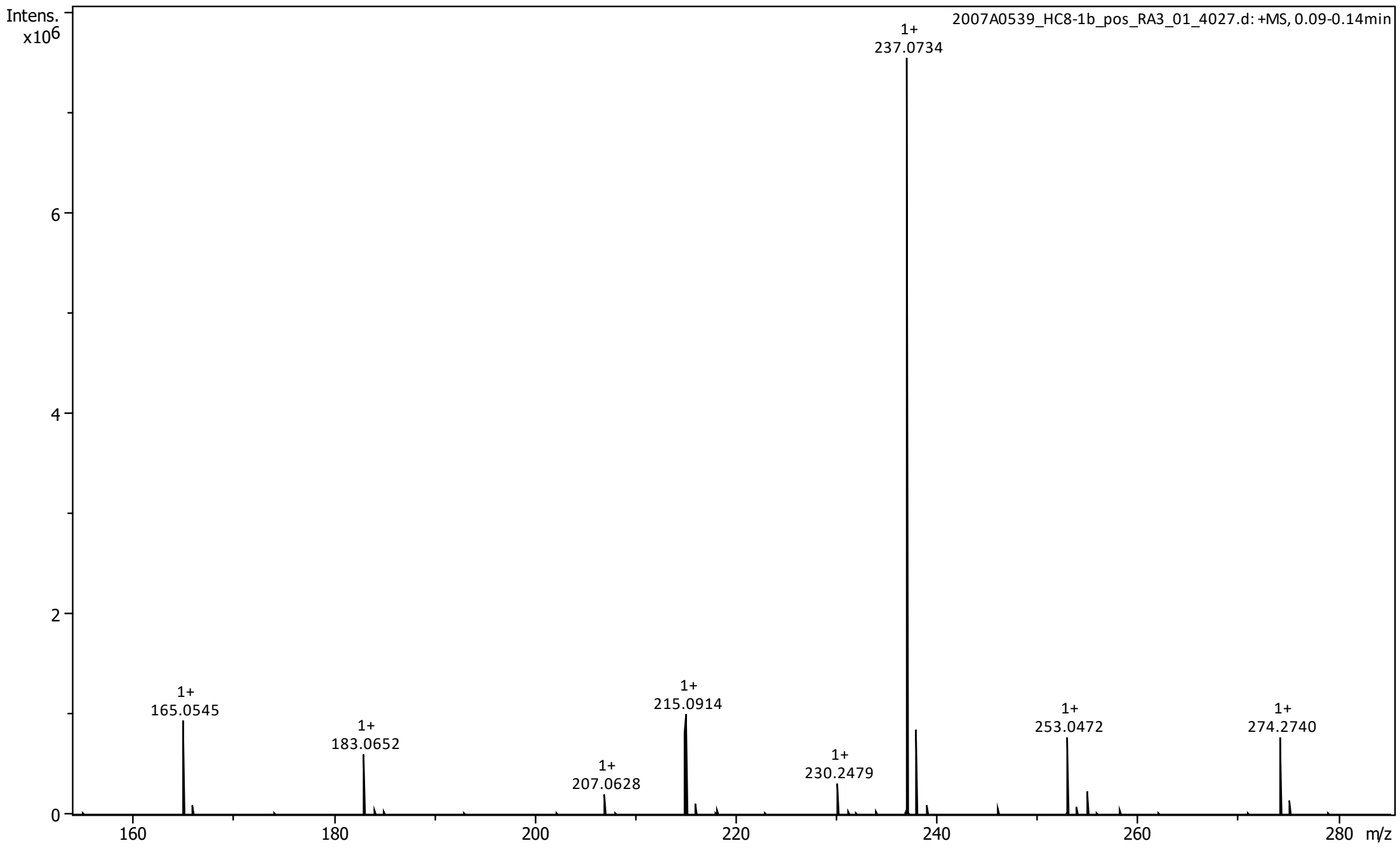

Figure. S19 HRESIMS spectrum of compound **3**.

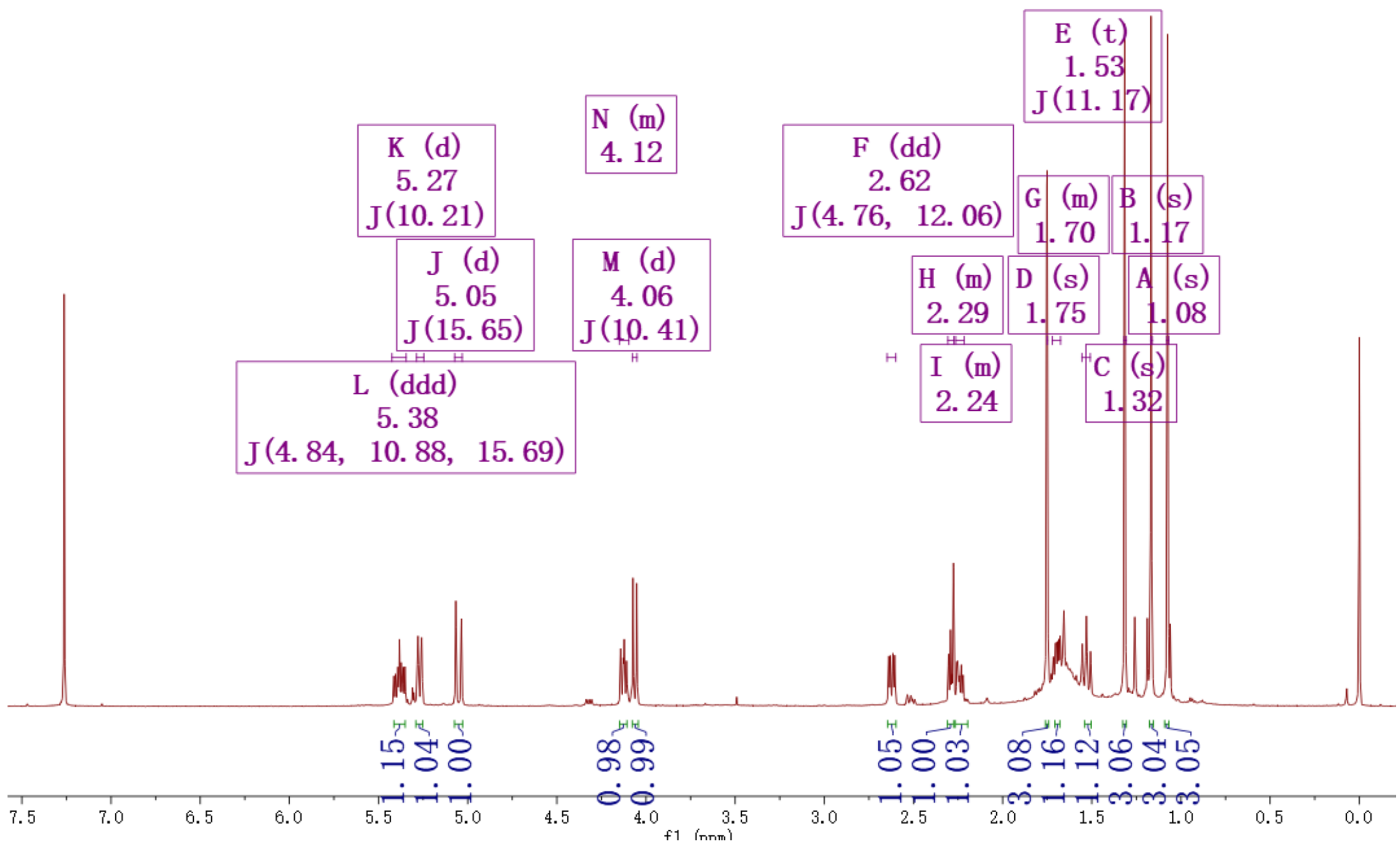

Figure. S20 ^{1}H NMR spectrum of compound **4** (500 MHz, $CDCl_3$).

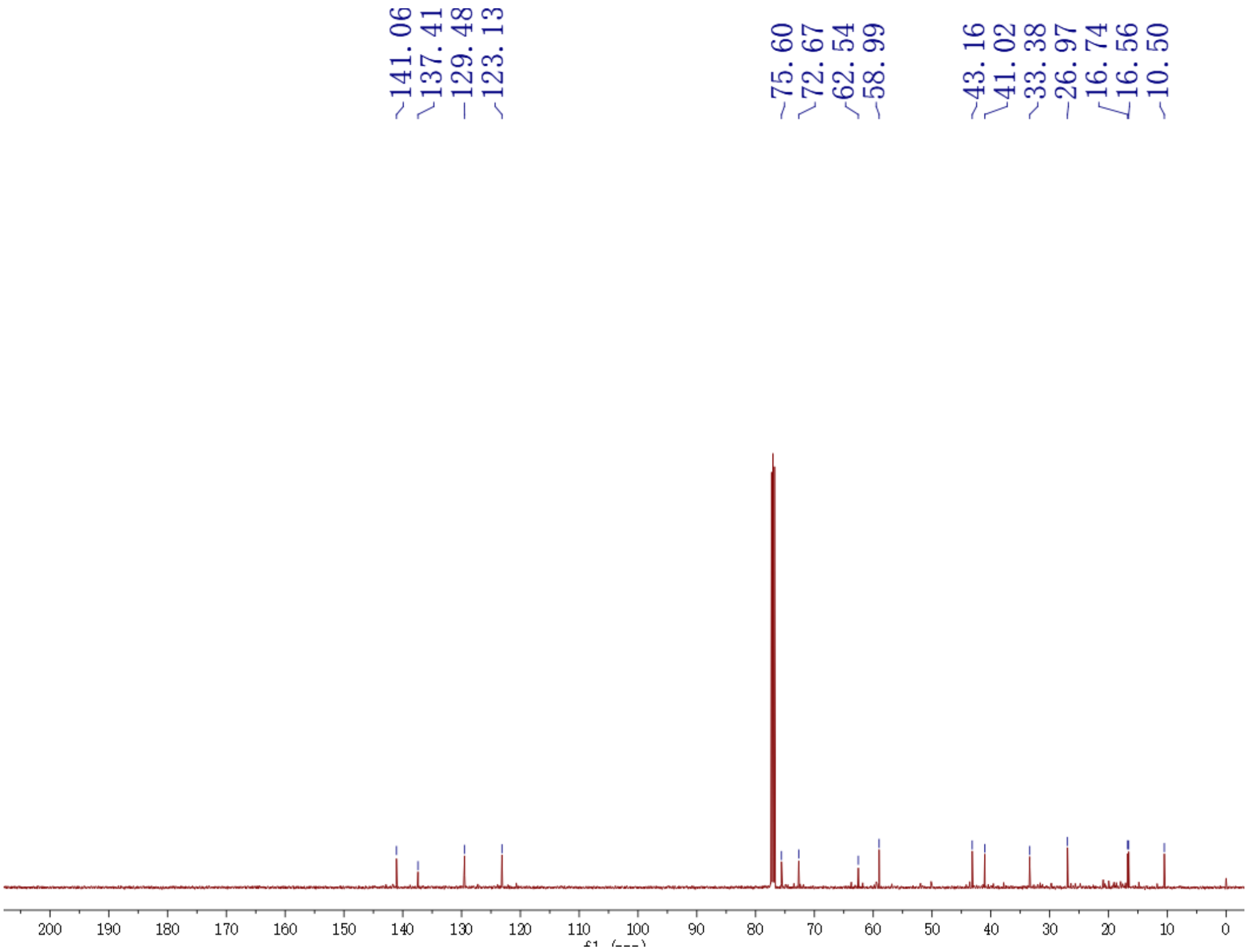

Figure. S21 ^{13}C NMR spectrum of compound **4** (125 MHz, $CDCl_3$).

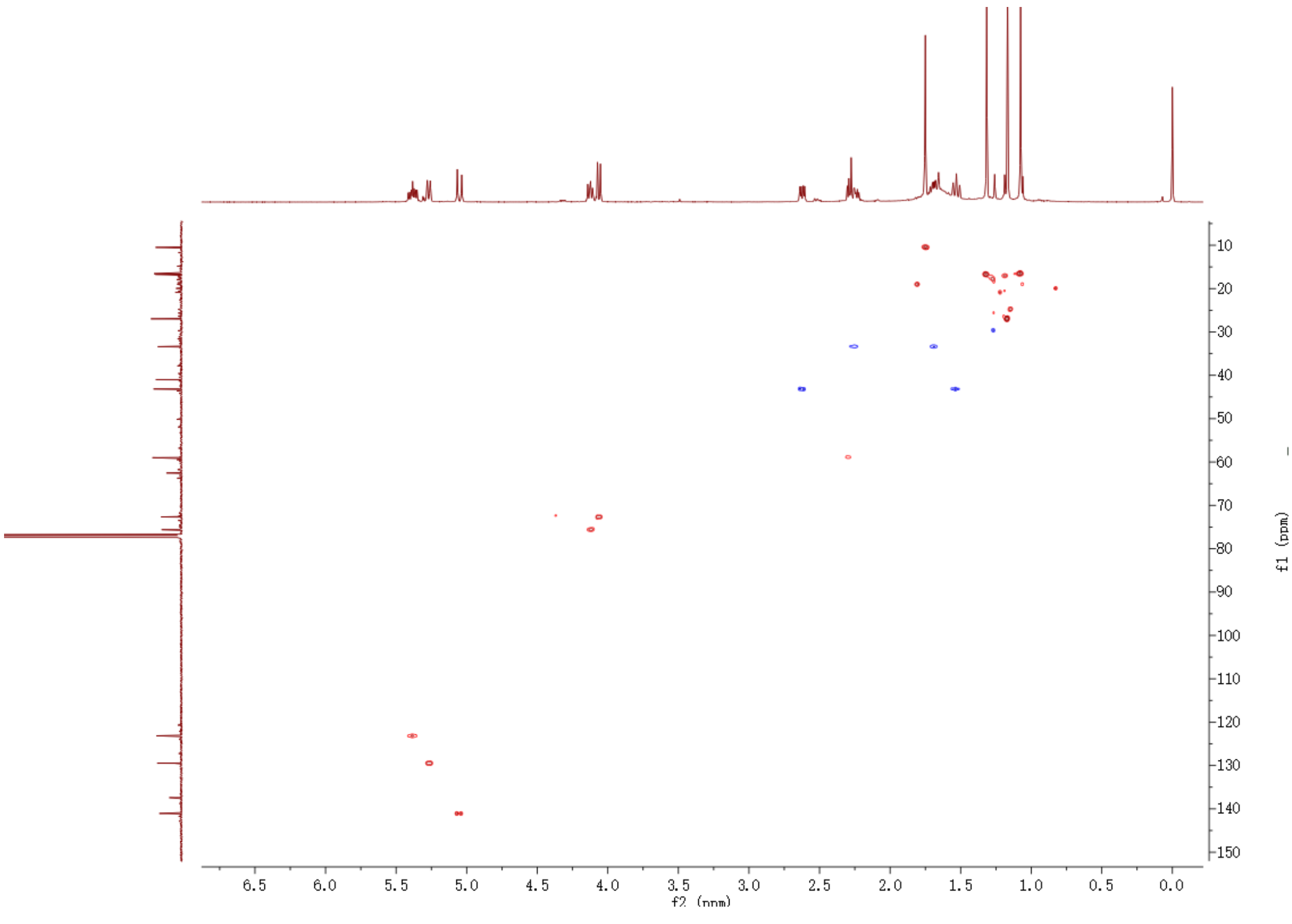

Figure. S22 HSQC spectrum of compound **4**.

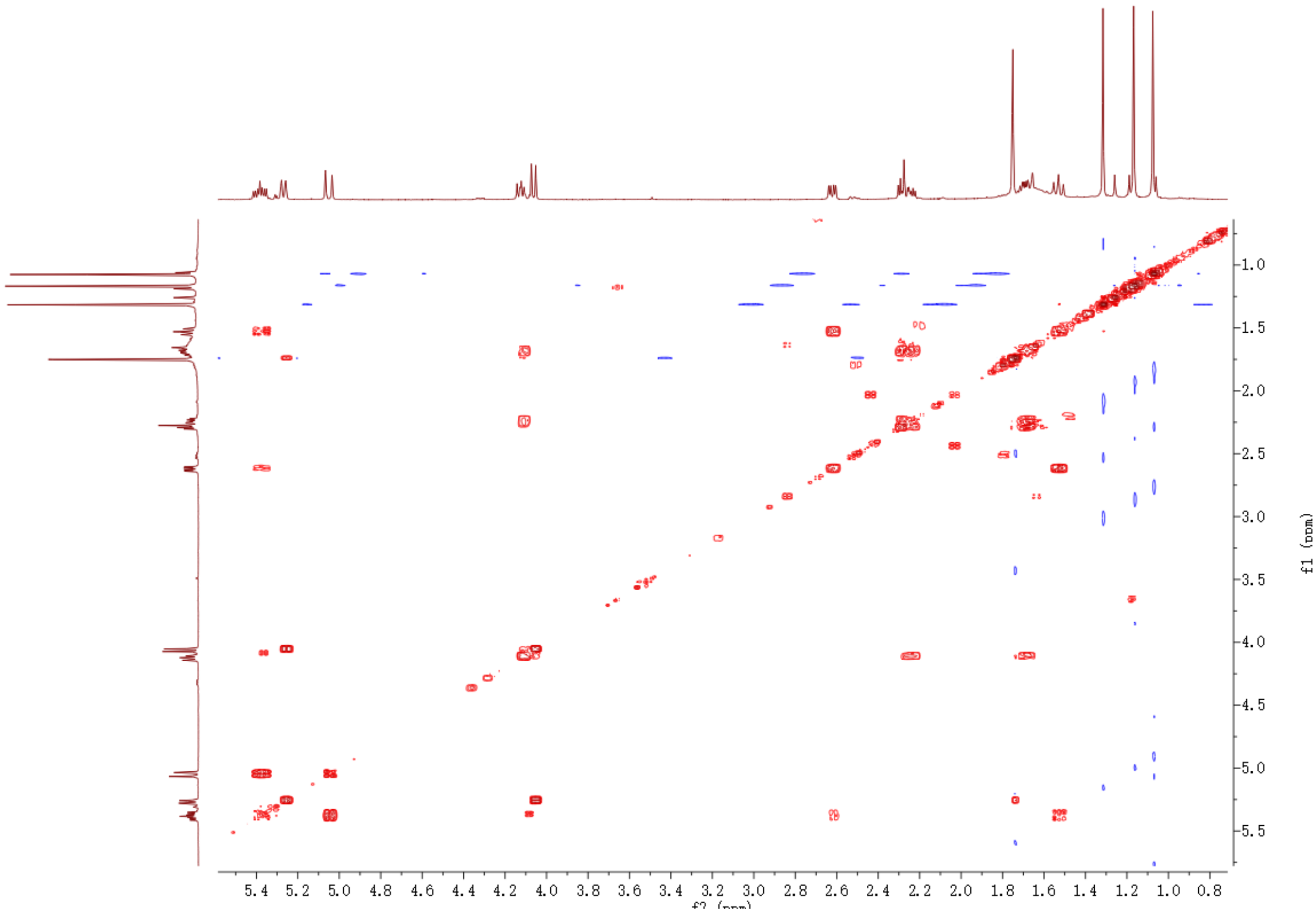

Figure. S23 ^{1}H-^{1}H COSY spectrum of compound **4**.

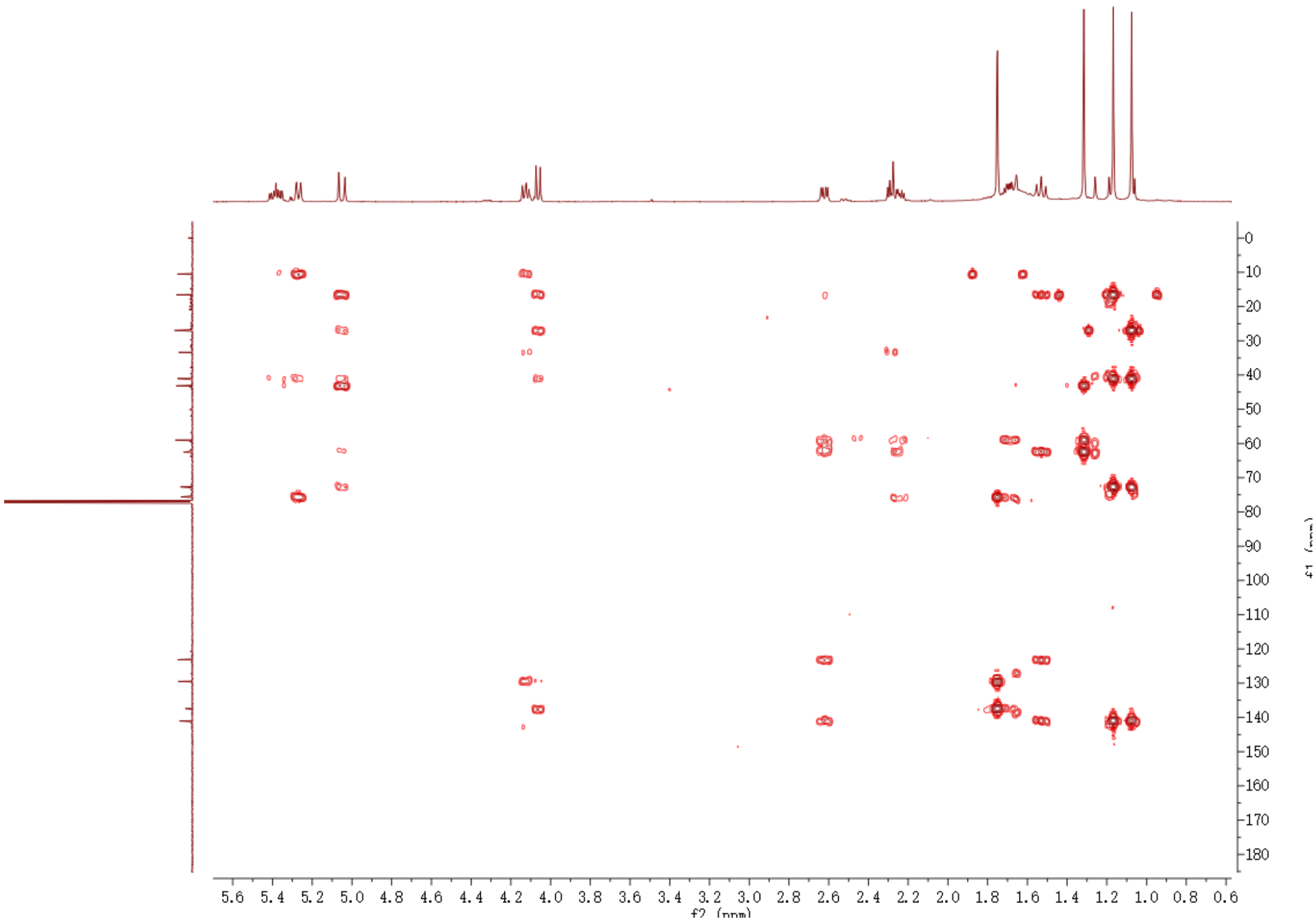

Figure. S24 HMBC spectrum of compound **4**.

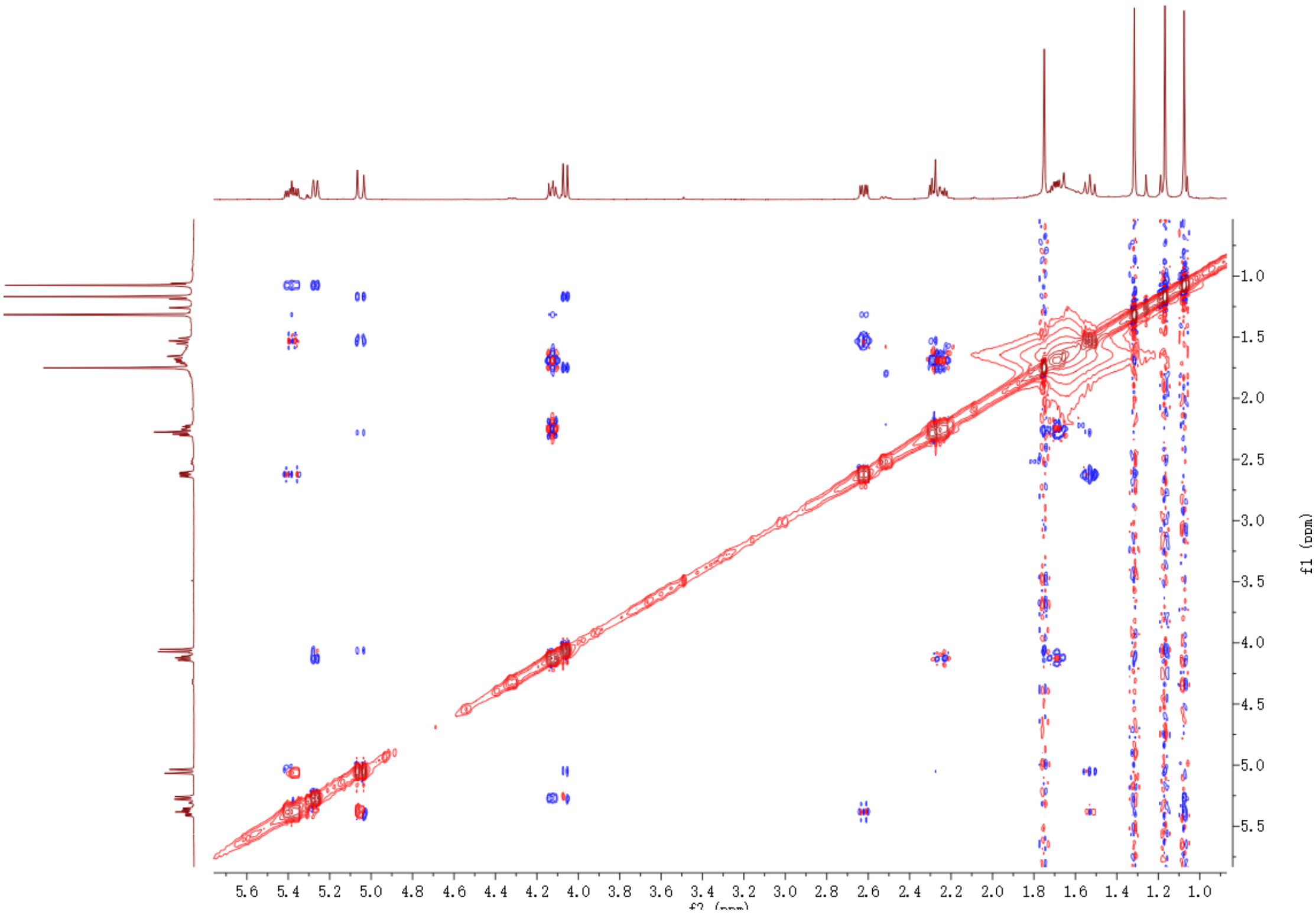

Figure. S25 HMBC spectrum of compound **4**.

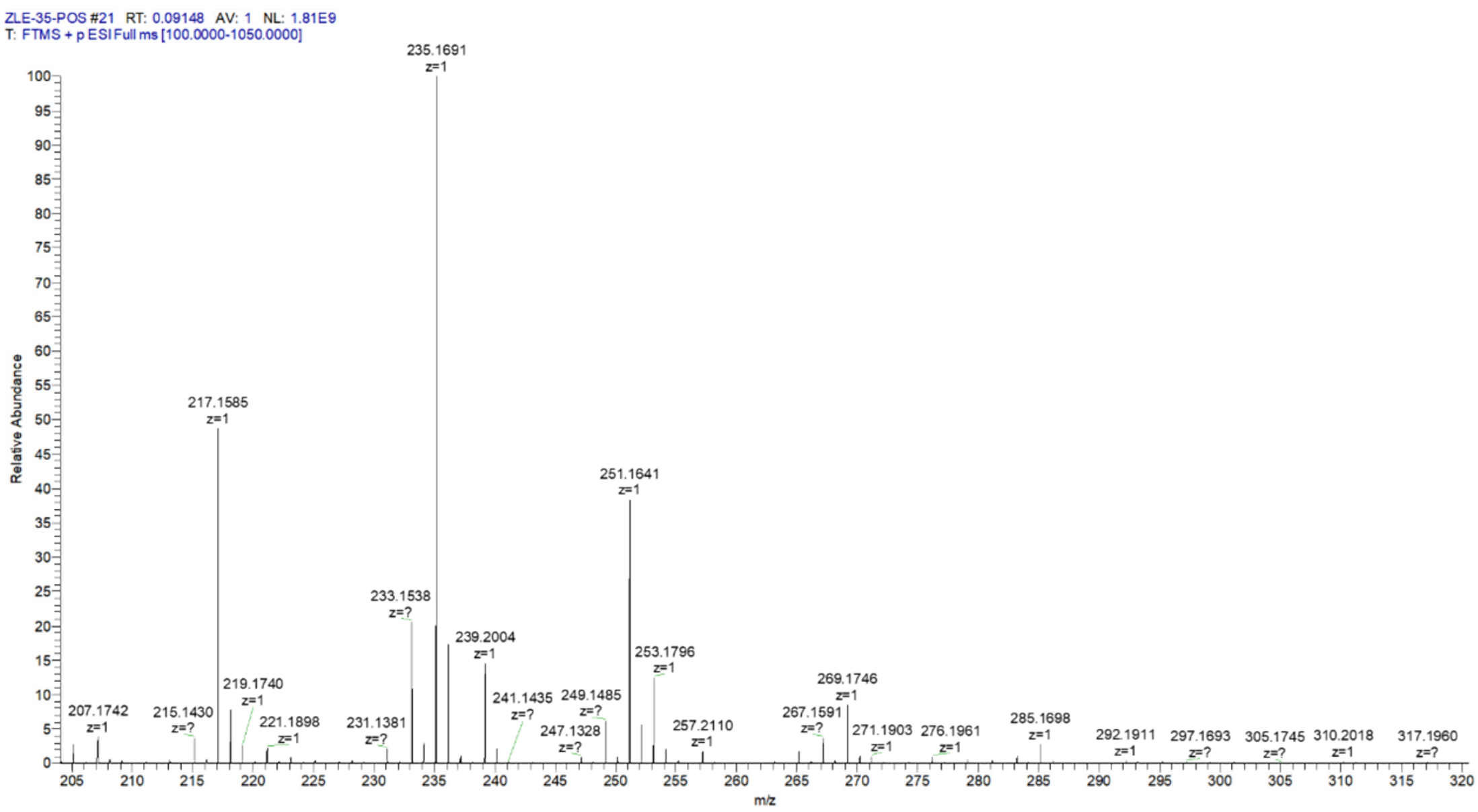

Figure. S26 HRESIMS spectrum of compound **4**.

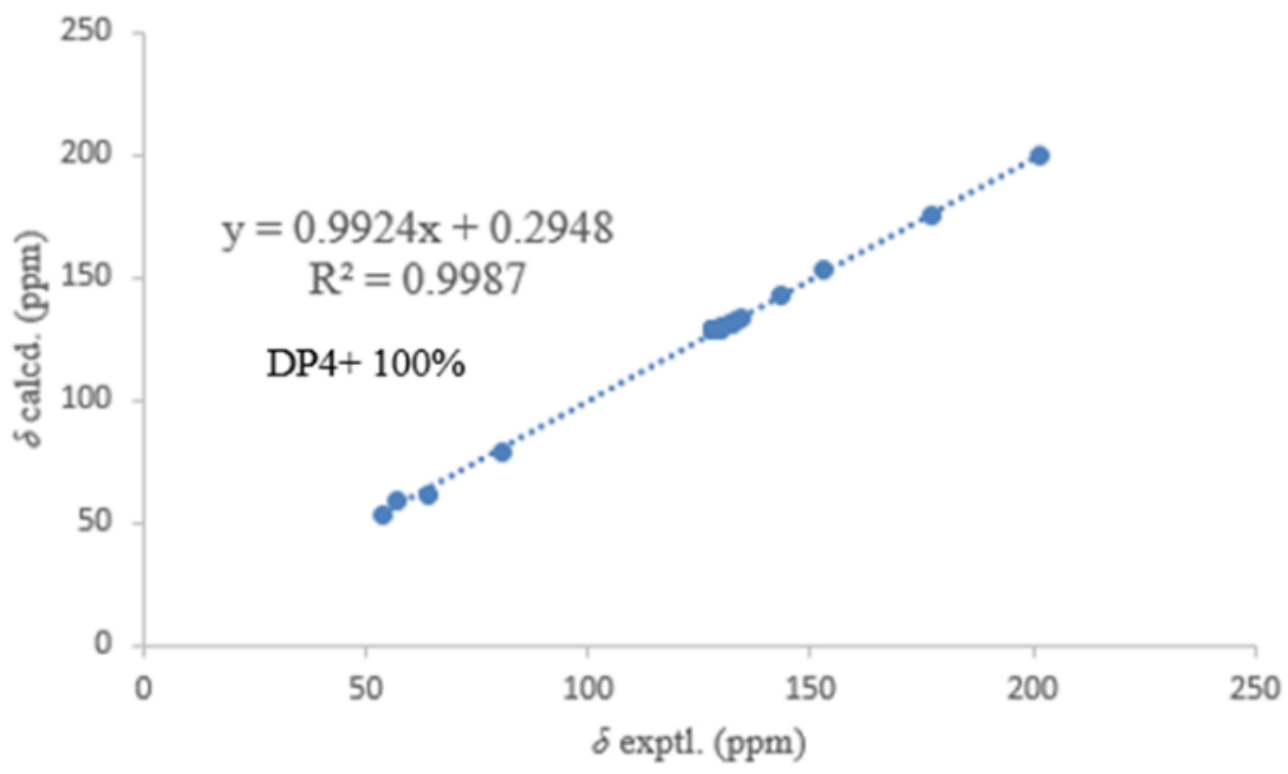

Figure S27. Comparison of calculated and experimental ^{13}C NMR data of **1**.

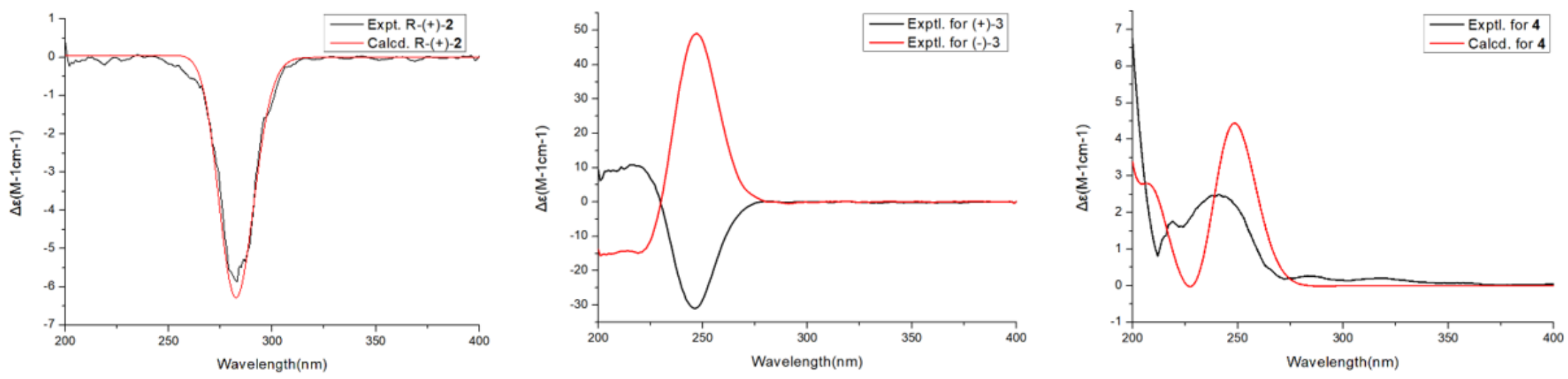

Figure S28. Experimental and calculated ECD spectra of compounds **2-4** in MeOH.

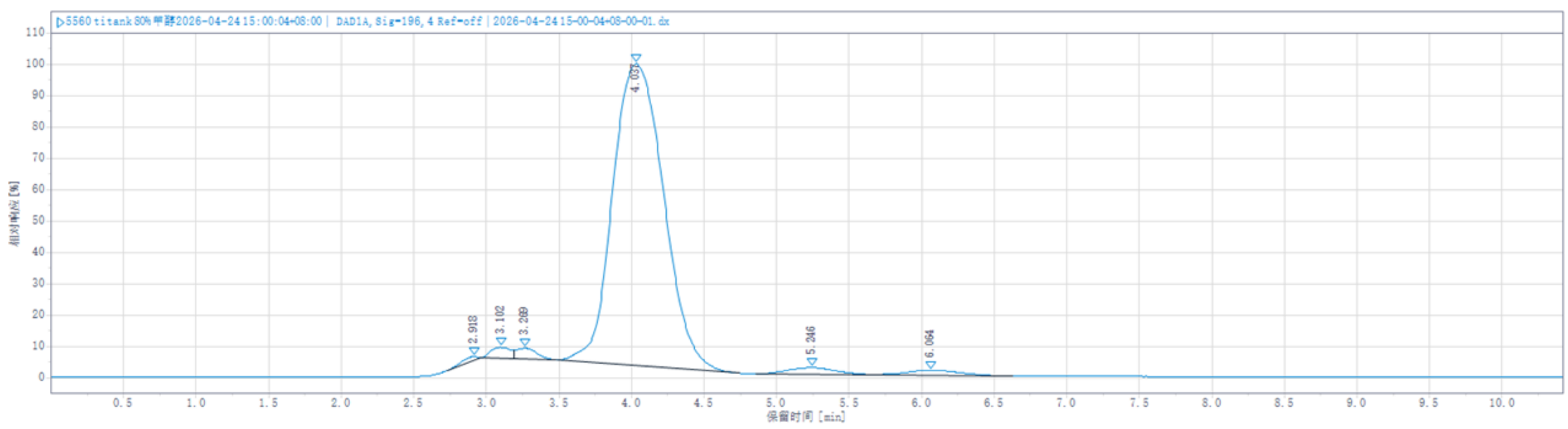

Figure 29. Chromatograms obtained from HPLC analyses of **2**.

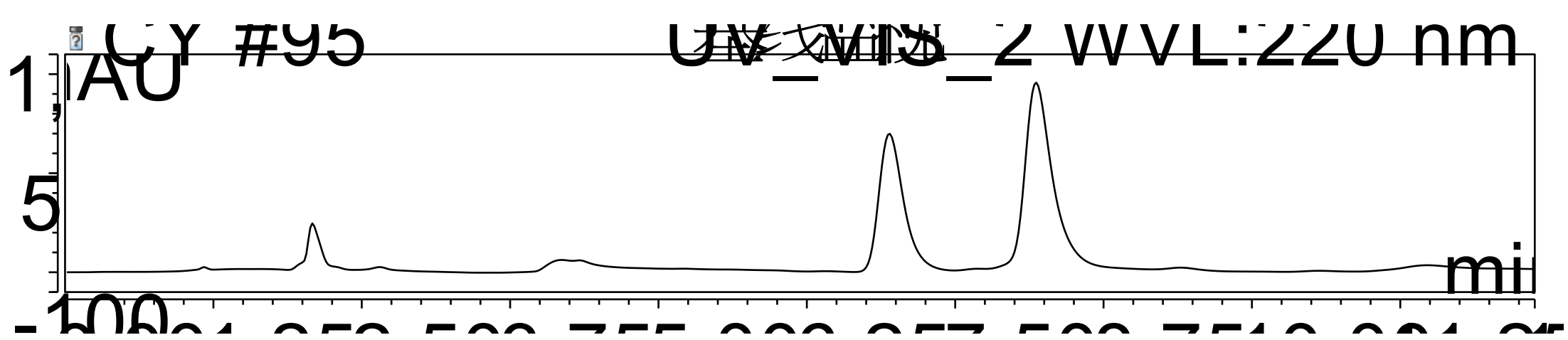

Figure S30. Chromatograms obtained from chiral HPLC analyses of **3**.

Table S1. The DP4+ evaluation of decemcellulin A (**1**).

Functional	Solvent?	Basis Set	Type of Data
mPW1PW91	PCM	6-31G(d,p)	Unscaled Shifts

		DP4+	100.00%	0.00%	–	–	–
Nuclei	sp2?	xperimenta	Isomer 1	Isomer 2	Isomer 3	Isomer 4	Isomer 5
C	x	142.7	143.2	146.3			
C	x	153.4	153.2	154.3			
C	x	199.3	201.8	198.9			
C		60.7	64.1	65.7			
C		78.7	80.7	85.5			
C	x	174.6	177.2	175.6			
C	x	131.8	133.2	131.8			
C	x	128.6	127.7	131.7			
C	x	128.7	128.3	127.1			
C	x	129.6	130.0	129.9			
C	x	128.4	127.8	127.5			
C	x	128.8	130.10	129.40			
C	x	132.9	134.70	133.70			
C	x	130.6	131.80	132.60			
C	x	128.6	127.70	128.50			
C	x	128.2	128.00	128.30			
C	x	128.6	128.20	128.30			
C	x	130.6	132.30	130.60			
C		58.8	57.20	57.60			
H		53.6	53.60	53.90			
H		4.01	4.00	4.21			
H	x	7.82	7.80	7.78			
H	x	7.4	7.40	7.43			
H	x	7.4	7.40	7.47			
H	x	7.4	7.49	7.47			
H	x	7.82	8.41	8.38			
H	x	7.15	7.19	7.46			
H	x	7.4	7.53	7.41			
H	x	7.4	7.44	7.44			
H	x	7.4	7.35	7.45			
H	x	7.15	7.19	7.21			
H		4.15	4.19	4.13			
H		3.77	3.55	3.28			

Functional	Solvent?	Basis Set	Type of Data
mPW1PW91	PCM	6-31G(d,p)	Unscaled Shifts

	Isomer 1	Isomer 2	Isomer 3	Isomer 4	Isomer 5	Isomer 6
sDP4+ (H data)	98.89%	1.11%	–	–	–	–
sDP4+ (C data)	99.92%	0.08%	–	–	–	–
sDP4+ (all data)	100.00%	0.00%	–	–	–	–
uDP4+ (H data)	99.16%	0.84%	–	–	–	–
uDP4+ (C data)	98.91%	1.09%	–	–	–	–
uDP4+ (all data)	99.99%	0.01%	–	–	–	–
DP4+ (H data)	99.99%	0.01%	–	–	–	–
DP4+ (C data)	100.00%	0.00%	–	–	–	–
DP4+ (all data)	100.00%	0.00%	–	–	–	–

Table S2 ^{1}H NMR (500MHz) and ^{13}C NMR (125MHz) data of compounds **2-3**.

	**2**			**3**	
no	δ_C[a], type	δ_H[a] (J in Hz)	no	δ_C[b], type	δ_H[b] (J in Hz)
1	169.7, C		1	106.7, CH	5.12, d (5.0)
2	137.9, C		2	42.6, CH	2.4, m
3	129.6, C		3	45.6, CH	2.70, dd (5.1,11.5)

4	78.0, CH	5.54, dd (1.5, 8.4)	4	172.5, C	
5	29.3, CH_2	2.44, m; 1.69, m	5	89.1, CH	5.15, s
6	28.6, CH_2	2.51, m	6	163.7, C	
7	175.2, C		7	102.1, CH	5.94, d (5.0)
8	130.4, C		8	11.5, CH_3	1.16, d (6.9)
9, 13	127.2, CH	7.79, d (7.4)	9	55.9, CH_3	3.45, s
10, 12	128.3, CH	7.47, t (7.1)	10	56.4, CH_3	3.80, s
11	128.4, CH	7.37, t (7.4)			

[a] Recorded in CD_3OD; [b] Recorded in $CDCl_3$.

Table S3 ^{1}H NMR (500MHz) and ^{13}C NMR (125MHz) data of compound **4** in $CDCl_3$.

	**4**			**4**	
no	δ_C, type	δ_H (J in Hz)	no	δ_C, type	δ_H (J in Hz)
1	76.5, CH	4.12, m	9	72.6, CH	4.06, d (10.4)
2	33.4, CH_2	2.23, m	10	137.3, C	
3	59.0, CH	2.30, m	11	129.5, CH	5.27, d (10.2)
4	62.6, C		12	16.7, CH_3	1.31, s
5	43.1, CH_2	2.62, dd (4.8,12.1)	13	26.9, CH_3	1.16, s
6	123.1, CH	5.38, ddd (4.8,10.9,15.7)	14	16.5, CH_3	1.07, s
7	141.0, CH	5.05, d (15.7)	15	10.5, CH_3	1.75, s
8	40.9, C				
