# Supplementary figures and images for: Metabolites with anti-inflammatory activity from the mangrove endophytic fungus Fusarium decemcellulare DQ-28
Source: RSC Adv. 2026 Jul 13. Online ahead of print. doi: 10.1039/d6ra05011e (PMC13358892; doi:10.1039/d6ra05011e)

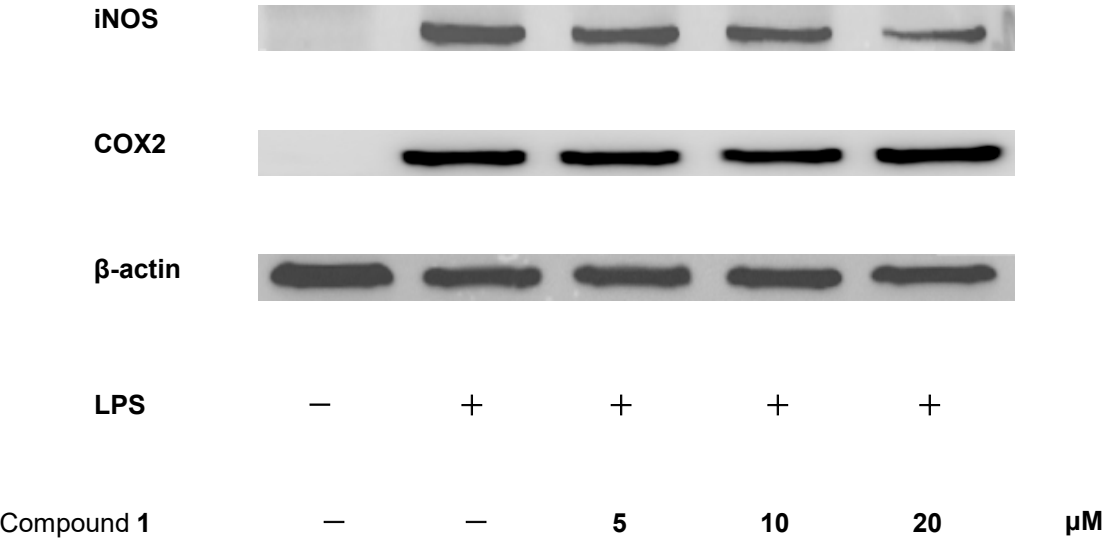

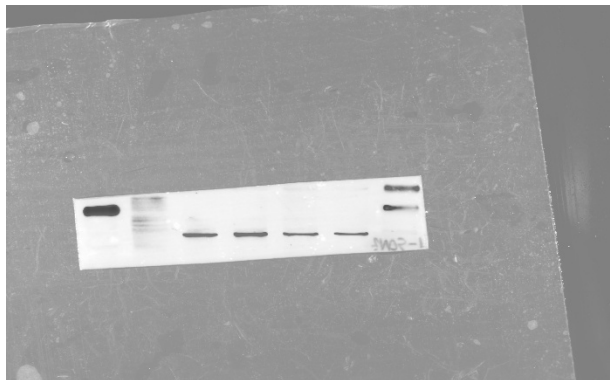

iNOS-1

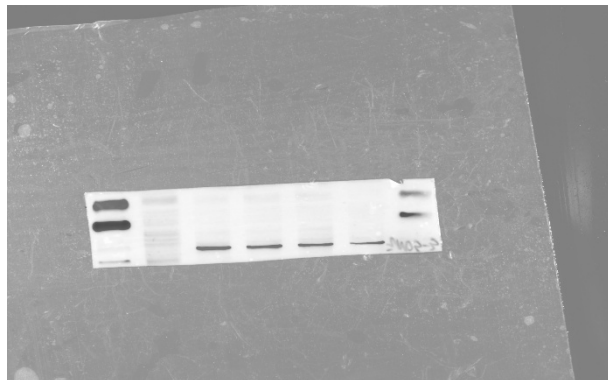

iNOS-2

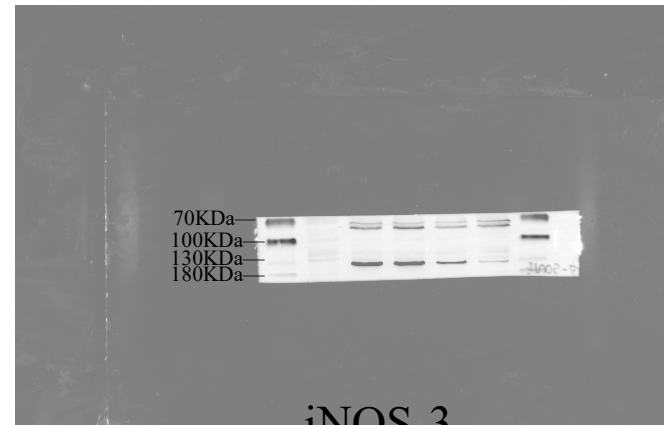

iNOS-3

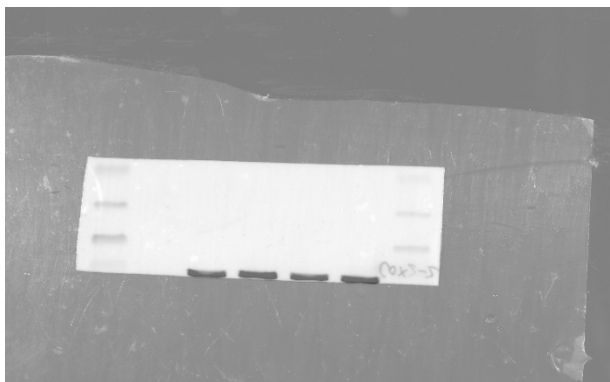

COX-2-1

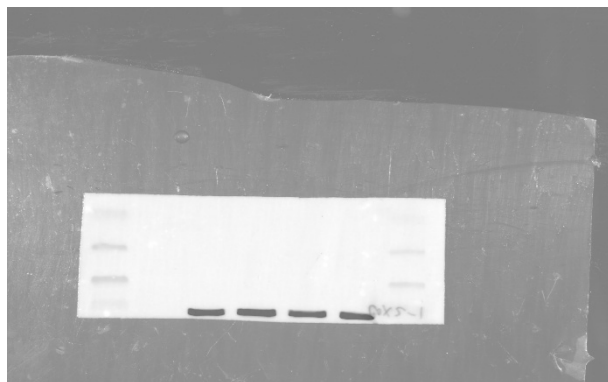

COX-2-1

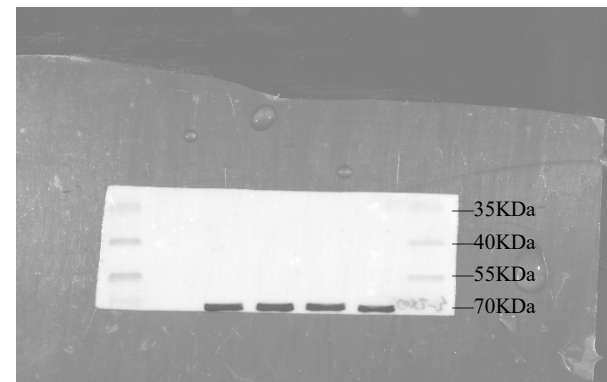

COX-2-3

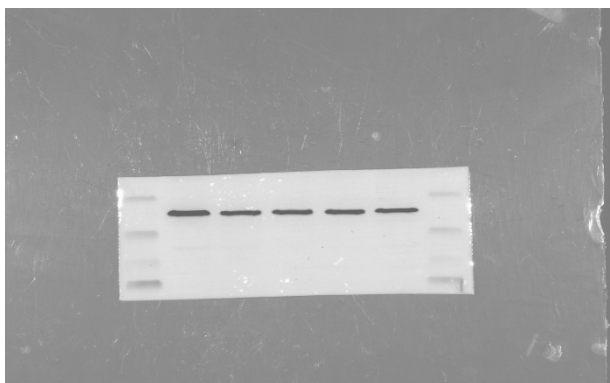

$\beta$ -actin-1

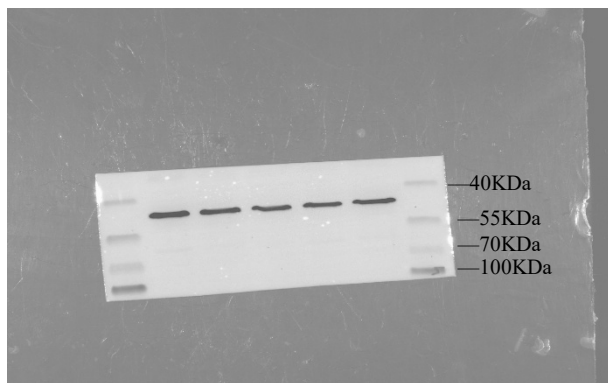

$\beta$ -actin-2

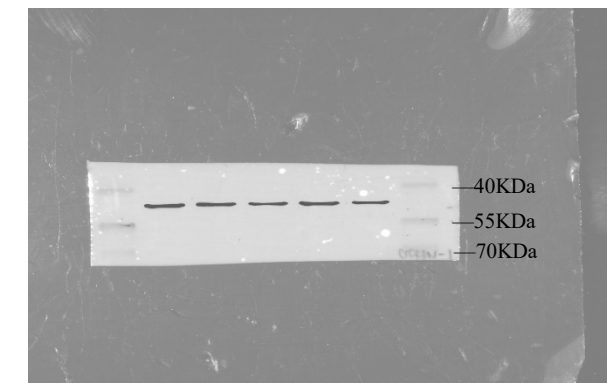

$\beta$ -actin-3

Supplement: RA-OLF-D6RA05011E-s001 [file RA-OLF-D6RA05011E-s001.pdf]
